# Sherlock: an open-source data platform to store, analyze and integrate Big Data for biology

**DOI:** 10.12688/f1000research.52791.1

**Published:** 2021-05-21

**Authors:** Balazs Bohar, David Fazekas, Matthew Madgwick, Luca Csabai, Marton Olbei, Tamás Korcsmáros, Mate Szalay-Beko

**Affiliations:** 1 Earlham Institute, Norwich Research Park, Norwich, UK; 2 Department of Genetics, Eotvos Lorand University, Budapest, Hungary; 3 Gut Microbes and Health Programme, Quadram Institute Bioscience, Norwich Research Park, Norwich, UK

**Keywords:** Software, Computational biology, Network biology, Systems biology, Data lake, big data, data management, data integration

## Abstract

In the era of Big Data, data collection underpins biological research more so than ever before. In many cases this can be as time-consuming as the analysis itself, requiring downloading multiple different public databases, with different data structures, and in general, spending days before answering any biological questions. To solve this problem, we introduce an open-source, cloud-based big data platform, called Sherlock ( https://earlham-sherlock.github.io/). Sherlock provides a gap-filling way for biologists to store, convert, query, share and generate biology data, while ultimately streamlining bioinformatics data management. The Sherlock platform provides a simple interface to leverage big data technologies, such as Docker and PrestoDB. Sherlock is designed to analyse, process, query and extract the information from extremely complex and large data sets. Furthermore, Sherlock is capable of handling different structured data (interaction, localization, or genomic sequence) from several sources and converting them to a common optimized storage format, for example to the Optimized Row Columnar (ORC). This format facilitates Sherlock’s ability to quickly and easily execute distributed analytical queries on extremely large data files as well as share datasets between teams. The Sherlock platform is freely available on Github, and contains specific loader scripts for structured data sources of genomics, interaction and expression databases. With these loader scripts, users are able to easily and quickly create and work with the specific file formats, such as JavaScript Object Notation (JSON) or ORC. For computational biology and large-scale bioinformatics projects, Sherlock provides an open-source platform empowering data management, data analytics, data integration and collaboration through modern big data technologies.

## Introduction

Most bioinformatics projects start with gathering a lot of data. Often bioinformaticians have to work on bespoke datasets, for example, gene expression or mutation data, but in almost all cases this requires some sort of external reference data. The vast majority of projects may call for genome annotations, gene ontologies, tissue-specific expression datasets, drug-related databases, and many other, particular reference datasets. One of the reasons why we use the term’bioinformatics’ in the first place is because we cannot correlate and process all these datasets manually, we need the help and the power of the computers and databases ( Greene et al. 2014). Thanks to the current technical advancement, many solutions exist worldwide to take advantage of the available computational possibilities ( Marx 2013).

Cloud storage solutions such as Amazon’s S3 ( https://aws.amazon.com/s3/) or Google Cloud Storage ( https://cloud.google.com/storage) offer scalability and flexibility to the matching compute solutions. More importantly, they allow large and potentially shared datasets to be stored on the same infrastructure where large-scale analyses are run. Some companies have utilized cloud resources to offer storage, access to shared datasets, and transparent sharing of data. Cloud storage also provides enhanced reliability, as the data is backed up in several geographical locations ( Kasson 2012). However, in most of the cases, these cloud storage companies only offer data storage solutions, but this does not include platforms to execute or work with data.

To address these issues, we repurposed concepts and open source tools from the top players of the software industry, like Facebook, Netflix and Amazon. With the aim to replace the tedious process of manual data collection before the first steps of any bioinformatics project, we developed a new platform, Sherlock. The Sherlock platform has two main parts: 1) a query engine, which is responsible for executing the given Structured Query Language (SQL) queries, which is the most extensively used database language; 2) a Data Lake, required for data storage, which consists of multiple different databases or datasets, where the Query engine can executes queries against the data. The combination of these two parts streamlines efficient data collection, data integration, and data preprocessing for a given project ( Khine & Wang 2018).

## Implementation and Operation

### Overview

Sherlock is an open source and freely accessible platform that combines several different software technologies together ( https://earlham-sherlock.github.io/). One of the core software that Sherlock uses is Docker. Docker is an open and widely used technology to isolate Linux processes and provide them a reproducible, well defined runtime environment independent from the actual operating system ( Matthias & Kane 2015). Each element in the Sherlock platform is running inside a separate Docker container. A Docker container is a standard unit of software that packages up all of the necessary code and all the dependencies for them, so the application runs quickly and reliably in an isolated environment on any computer. Container orchestration tools are used when a complex solution (like Sherlock) requires the interconnection of many docker containers distributed among multiple separate Linux machines. In Sherlock, we are using the standard Docker Swarm orchestration platform (Smith 2017), as it is easy to use and much easier to operate than other orchestration frameworks (like Kubernetes ( https://kubernetes.io)). These industry standard technologies make the management (starting, stopping and monitoring) of Sherlock easy, while also enabling us to implement more advanced features, for example the on-demand scaling of the analytical cluster where Sherlock is running. This scalability is the biggest advantage of Docker Swarm. The entire cluster can be shut down or reduced to the minimum in the cloud when it is not used, while it can be scaled up to hundreds of nodes if necessary for executing very complex analytical queries. Other key benefits associated with Docker Swarm is the high level of availability offered for applications. This has a hierarchical structure, where several worker nodes and at least one manager node is responsible for handling the worker nodes' resources and ensuring that the cluster operates efficiently.

**Figure 1.**
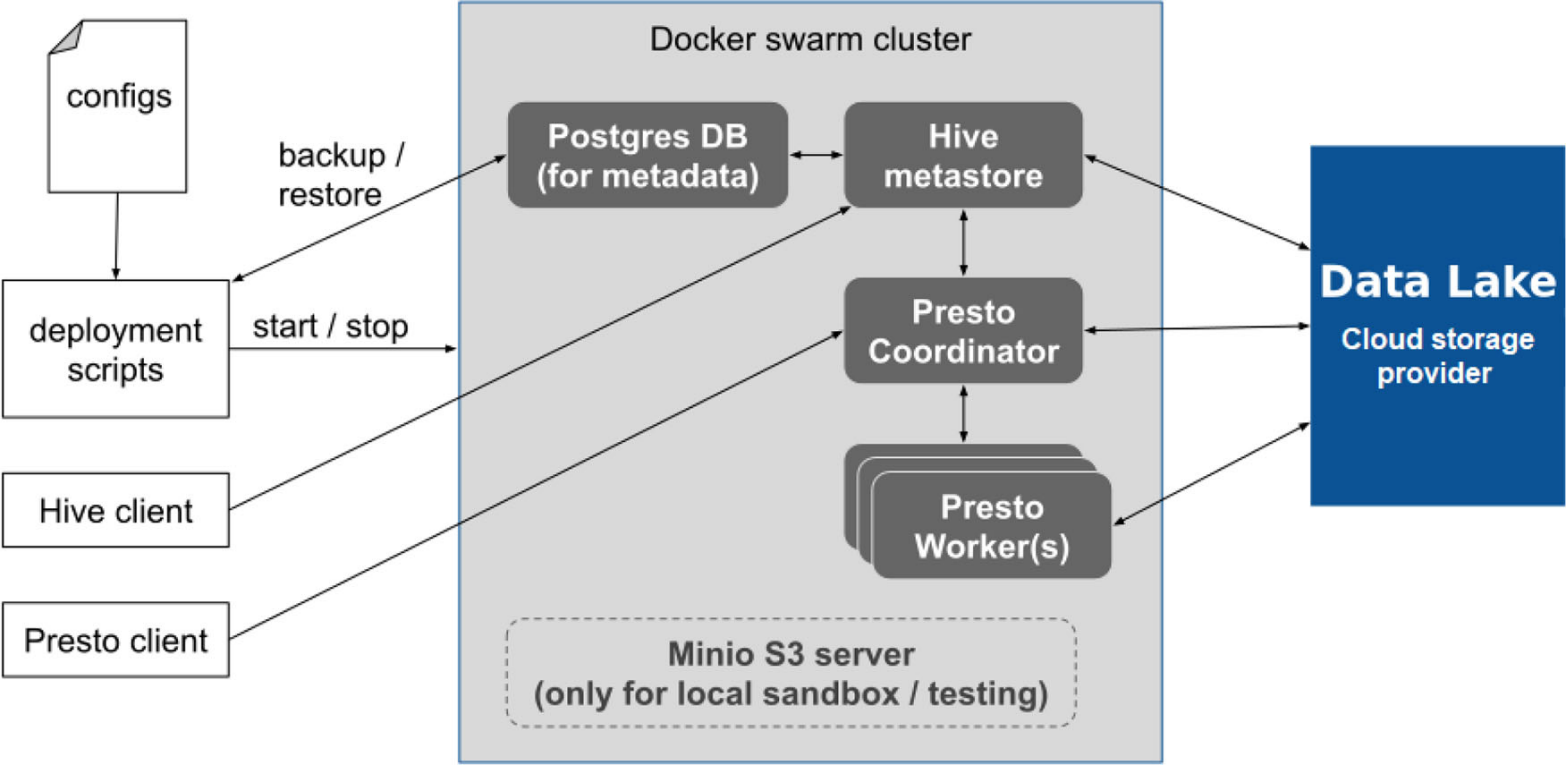
Overview of the Sherlock platform and its relationship with the Data Lake. In this image you can see how the different tools and the deployment scripts are connected to each other inside the core of Sherlock. The blue box represents the Data Lake, where the data is stored. You can see that only the Hive Metastore, the Presto Query Engine and the possible worker nodes have connection to the Data Lake. The box with the dashed line shows the Minio S3 server, where for example, the Sherlock platform can be used to test various features available in Sherlock.

The first step for using Sherlock is to start a Docker Swarm with the deployment scripts on a cluster with several worker nodes. These scripts start different services, but each in a separate container. These deployment scripts are configurable ( Figure 1). One can specify exactly how many resources a service can use at once (e.g. CPU, memory allocation). Inside a running Docker Swarm, there are two different main parts of Sherlock: the Hive metastore and the Presto query engine. Sherlock follows the industry best practices in its architecture to enable scalability. Usually, the main idea behind scalable batch processing architectures is the separation of data storage and analytics ( Dean & Ghemawat 2008). This architecture allows us to scale the analytical power and the storage size independently from each other and even dynamically. This is the case with Sherlock when deployed in the cloud.

The Sherlock platform does not include data, instead provides a platform where one can work on and manipulate the data ( Figure 1). Then by leveraging powerful database architectures (Presto query engine and Hive metastore) and providing them a channel to connect to the Data Lake (where we are storing our data) the user can run different analytical queries on top of the data files. The starting step for using Sherlock is to submit an SQL query to the Presto query engine, which can answer the biological question in interest and then it will connect to the Data Lake and fetch the necessary data files. Then the final step is to execute the submitted SQL query on top of the data files and then it takes back the results to the user ( Figure 1).

The minimal requirements for Sherlock depend on what the use case is and where Sherlock will be deployed. Sherlock can be used on a single laptop, single virtual machine (VM) or on a distributed cluster. Regarding using Sherlock on a single laptop we recommend using a relatively powerful laptop, which means that it has at least a dual-core processor (CPU) and at least 12 GB memory (RAM), as around half of these resources can be consumed by Sherlock. But the minimum requirement is at least 8 GB RAM and a dual-core processor. On the other hand, if the user wants to use it on a cluster with nodes, we will assume to have at least two machines with 32 GB memory (RAM) for each, and each of them having 8 processor (CPU) cores, but this can be scaled down to 16 GB RAM and 6 CPU cores.

### Sherlock as a platform

*Query engine*

**Figure 2.**
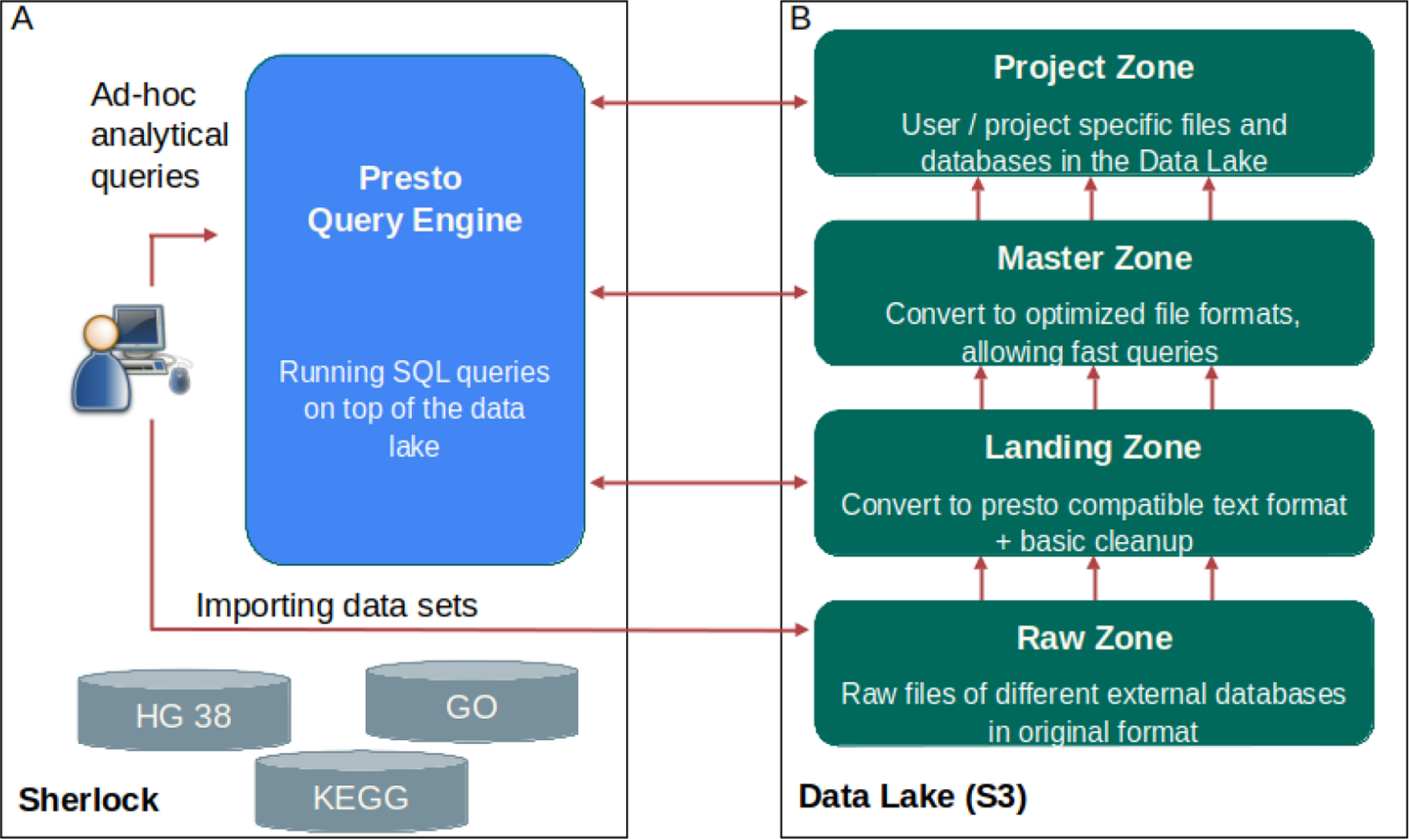
The relationship between the two parts of Sherlock: the platform itself with the query engine and the Data Lake. **A**: The structure of Sherlock data platform with the core part, which is the Presto Query Engine. The user can execute different analytical SQL queries with the help of this query engine. Presto will run these queries on top of the data files, which are inside the Data Lake (B) **B**: The structure of our Data Lake. You can see the four different zones inside the Data Lake, what we are using right now. 1) The raw zone with the raw data from the different external databases. 2) The landing zone, with the Presto compatible text files in JSON Lines format. 3) The master zone, where the data is in a detailed and exact format, called ORC. 4) The project zone, where we store only the data which is needed only for specific projects.

The first core part of Sherlock is the Query Engine ( Figure 2A), which is responsible for running the given question through SQL commands on top of the data files and retrieving the ‘answer’ for the user. A Query Engines, for example Hive, Impala, Spark SQL or Presto, are distributed, mostly stateless and scalable technologies. In our case, we are using the Presto Query Engine ( https://prestodb.io), developed by Facebook. Presto provides a high-performance Query Engine wrapped in an easily deployable and maintainable package. Furthermore, the Presto Query Engine can handle many different types of data storage solutions.

With Presto, we can formalize analytical questions using SQL. SQL is composed of three main sub-languages: 1) data definition language (DDL), which allows the specification of database schemas; 2) a data manipulation language (DML), which supports operations on data (store, modify, delete; 3) and a data control language (DCL), which enables database administrators to configure security access to databases. On the one hand SQL is designed for working data, which held in a relational database management system (RDBMS), on the other hand it can be used for stream processing in a relational data stream management system (RDSMS). Moreover it is particularly really useful to handle with structured data ( Silva et al. 2016). When combined with a Query Engine, SQL can be used to connect to the Data Lake, read and combine the data stored within it and execute different analytical queries, either sequentially or in parallel depending on the power of the given computer.

*Hive metastore*

The second core part of Sherlock is the Hive metastore ( https://docs.cloudera.com/runtime/7.0.1/hive-metastore/topics/hive-hms-introduction.html), which is a service that stores metadata related to Apache Hive and other services, in a backend RDBMS, such as MySQL or PostgreSQL. With regards to Sherlock, Presto stores the metadata about the folders in the Data Lake and the Hive metastore which contains only the meta information about the different folders, which is in the Data Lake. In Sherlock we provide simple scripts to save this metadata in the Data Lake when you want to make a backup or before you want to terminate your analytical cluster.

### Deployment

For Sherlock, we developed a dockerized version of Presto that also incorporates the Hive metastore. This addition of both the query engine and metastore makes Sherlock cloud-agnostic, so it can be installed at any cloud provider, providing it is a Linux machine with Docker installed. The advantage of running Presto in a dockerized environment is that it is not necessary to install and configure the whole Presto Query Engine manually. Furthermore, it can even be fired up on a local machine, multiple machines or any cloud service as well. On the deployment guide section of the GitHub page of Sherlock ( https://earlham-sherlock.github.io/docs/deployment_guide.html), we show how to make a set of Linux virtual machines with Docker installed, then start a distributed Presto cluster by using the Sherlock platform.

### The Data Lake - data storage

A Data Lake is a simple network storage repository that can be accessed by external machines ( https://aws.amazon.com/big-data/datalakes-and-analytics/what-is-a-data-lake/). All the data which is imported into, transformed in, or exported from Sherlock will be stored here as simple data files. The biggest advantages of using a Data Lake is that its operation will be the same on a local machine, on the cloud and the data can be stored in a well structured way on a reliable storage. Furthermore it can be scaled independently from the query engine. The technologies in Sherlock are compatible with both of the most common Data Lake solutions; Hadoop Distributed File System (HDFS), which is a distributed file system designed to run on commodity hardware, and with the Simple Storage Service (S3) storage formats. However, we decided to only describe S3 in all of our examples, as S3 is more widely-used, modern, and accessible. Although HDFS is an older standard solution, it has some very powerful features which can result in better performance, but all in all it is also much more difficult to set up, maintain and in most cases its extra features are not necessary for the given project. In contrast, S3 is a standard remote storage API (Application Programming Interface) format, introduced first by Amazon ( https://aws.amazon.com/s3/). As of writing, you can purchase S3 storage as a service from all major cloud providers like Digital Ocean, Amazon AWS, Google Cloud or Microsoft Azure and each of these can be compatible with the Sherlock platform.

Having somewhere to store the data is only one half of having an operational Data Lake. One cannot stress enough how important it is to organize the data in a well defined ‘folder’ structure. Many Data Lake deployments become unusable after a few years due to poor maintenance, resulting in a large amount of data ‘lost’ in the thousands of folders, or inconsistent file formats and no ownership over the data. Our solution is to separate all of the data in the Data Lake into four different main folders, representing different stages of the data and different access patterns in the Data Lake. Inside each of these main folders we can create subfolders, and it is a good practice to incorporate the name of the dataset, the data owner name, the creation date (or other version info), and the file formats somehow into their paths.

**Figure 3.**
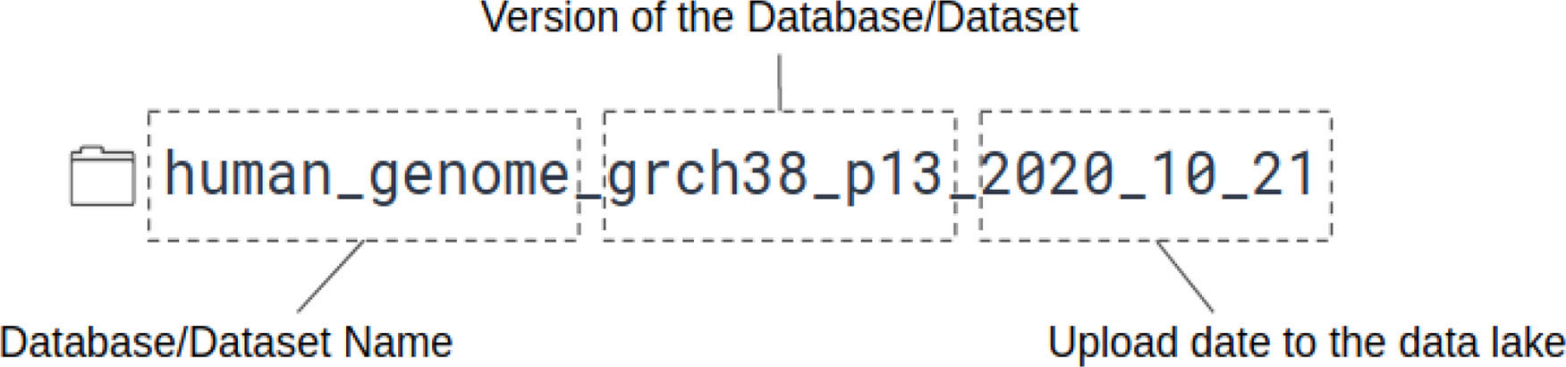
An example strategy, how one can store data inside the Data Lake. The first part of the folder name is the name of the given database/dataset. In that case the human genome. The next part is the version of the data and the last is the uploaded date to the Data Lake.

We separated our Data Lake into four different main zones, which are built on top of each other ( Figure 2B). The first zone is the raw zone. Into the raw zone, we archived all the database files in their original formats. For example: if we downloaded the human genome, then we put the fasta files here, under a separate subfolder. The name of the subfolder should contain the exact version (for example: hg38_p12) ( Figure 3), and also we put a small readme file to the folder, where we listed some metadata, like the date and the url of the download, etc. Usually, these files cannot be opened with Presto, as the file format is incompatible in most of the cases.

The next zone is the landing zone. We needed to develop specific scripts, called loader scripts, converting and extracting the raw datasets into this zone. We converted the data to a text file in JSON Lines format ( https://jsonlines.org), which can be opened by Presto. This is a specific JSON format, where each line of the text file represents a single JSON record. Each JSON file for a given dataset is placed into a separate sub-folder in the landing zone. It is then registered by Presto which sees the dataset now as a table. Then, Presto will be able to load the data, and perform other operations, for example it can execute simple data transformations on the data. However, we do not recommend using the tables in this zone for querying, because processing the large JSON files is very slow.

Using Presto, we converted the data from the landing zone into an optimized (ordered, indexed, binary) format to the next zone, which is the master zone. The main idea is that we use the tables in the master zone later for analytical queries. Here the data is in a more detailed and exact format, called Optimized Row Columnar (ORC), which is a free and open-source column-oriented data storage format ( https://orc.apache.org/docs/). With ORC, Sherlock can perform SQL queries much more faster than using the JSON text file format from the zone below. If necessary, advanced bucketing or partitioning on these tables can be used to optimize the given queries. Furthermore, the master zone contains the ‘single source of truth’. This means that the data here cannot be changed, only extended upon, for example adding a new version of the datasets.

The last zone is the Project zone. This is where we save the tables that are needed only for specific projects. We can even create multiple project zones, one for each group, project or user. It is important to have a rule to indicate the owner of the tables, as mentioned before this dramatically increases the effectiveness of Sherlock and ease of maintenance.

### Functionality of Sherlock

**Figure 4.**
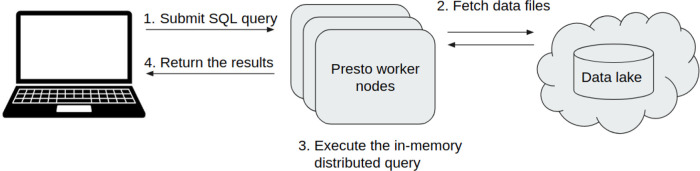
Overview of how the query engine and the Data Lake work together. This image represents the different steps, how a user can submit an analytical SQL query and what is the real path of this query until the results come back to the user.

Here, we describe the key steps of how the query engine and the Data Lake can be used together ( Figure 4). The first step is to submit an SQL query to the Presto query engine, then it will connect to the Data Lake and fetch the necessary data files. The third step is to execute the in-memory distributed query and then it takes back the results to the user.

It is important to mention that Sherlock has been designed for biologists, especially for network and system biologists. It can contain specific, interaction, expression and genome data-related databases thanks to loader scripts that are able to create the specific file formats from the source databases and upload them into the Data Lake. With these individual loader scripts, users can make and work with the necessary file formats from the different data sources without major time and coding efforts with their own scripts. The whole source code of the platform and the loader scripts ( Table 1) are freely available on Github: https://github.com/earlham-sherlock/earlham-sherlock.github.io

**Table 1.** The specific loader scripts, which are already included in Sherlock’s github repository.

Datatype	Loader script	Source	Reference
**General datasets**	DBSnp database	https://www.ncbi.nlm.nih.gov/snp/	( Smigielski et al. 2000)
	Gene Ontology	http://geneontology.org	( Ashburner et al. 2000)
	Gene Ontology Annotations		
	Human Genome	https://www.gencodegenes.org	-
	Uberon Gene Ontology	https://www.ebi.ac.uk/ols/ontologies/uberon	( Mungall et al. 2012)
	Uniprot ID Mapping data	https://www.uniprot.org	( UniProt Consortium 2021)
**Interaction databases**	BioPlex database	https://bioplex.hms.harvard.edu	( Huttlin et al. 2020)
	Dorothea database	https://dorothea.opentargets.io/#/	( Garcia-Alonso et al. 2018)
	HINT database	http://hint.yulab.org	( Das & Yu 2012)
	HuRI database	http://www.interactome-atlas.org	( Luck et al. 2020)
	InBioMap database	https://inbio-discover.com	( Li et al. 2017)
	IntAct database	https://www.ebi.ac.uk/intact/	( Orchard et al. 2014)
	IRefIndex database	http://irefindex.uio.no	( Razick et al. 2008)
	Mentha database	https://mentha.uniroma2.it	( Calderone et al. 2013)
	Omnipath database	https://omnipathdb.org	( Türei et al .)
	STRING database	https://string-db.org	( Szklarczyk et al. 2019)
**Expression data**	Bgee database	https://bgee.org	( Bastian et al. 2021)

In the next section, we will outline three different use cases on how Sherlock can be used and what we can use it for.

## Use Cases

### Use Case 1: Identifier (ID) mapping

One of the most crucial and common tasks for those working in the field of bioinformatics is ID mapping. It is difficult to work with many different datasets from many different sources, all carrying diverse identifiers. In addition, these identifiers can have clashing structures, which makes it complicated and time consuming to work with them. Users have to make different scripts or use different tools to work with these identifiers at once. Nowadays the main and key idea behind these ID mapping steps is to have a separate table or tables, called mapping tables, which contain the different identifiers, but only those. The best practice is to have only one mapping table which contains all of the necessary identifiers for a given project. The limitation with this approach is when a team has to work with so many different identifiers at once means that this mapping table can be really large, and it can take a lot of time to go through it and get the data, which is needed.

In Sherlock, users can work with just one mapping table, which can be made easily with the help of the provided loader scripts from the Github repository, and it can significantly shorten the time needed for ID mapping. Sherlock can execute these queries very quickly despite the large size of the mapping tables, owing to the implemented ORC format. To demonstrate this capability in Sherlock, we search for genes from the STRING database ( Szklarczyk et al. 2019), which are expressed only in the brain.

**Figure 5.**
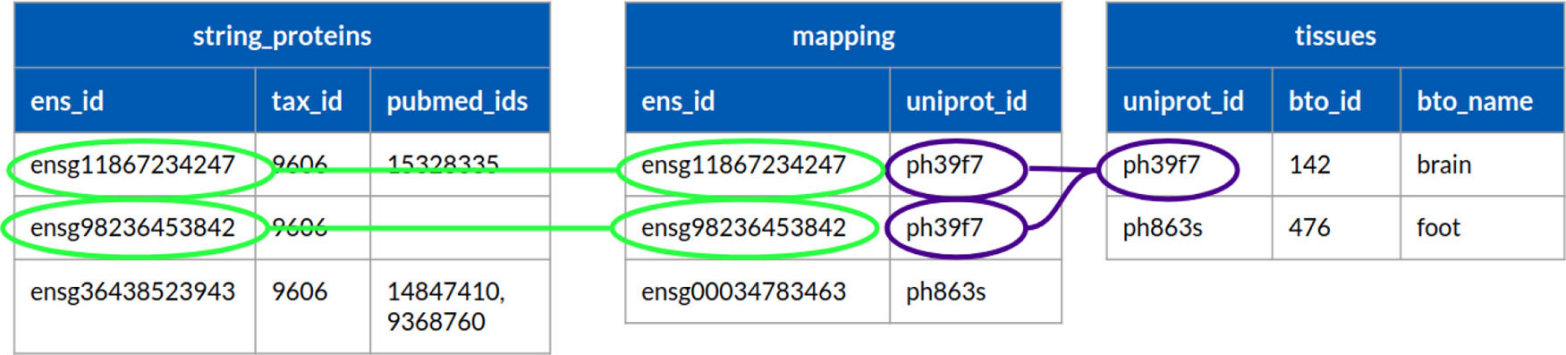
Example query, how can we map between different identifiers with Sherlock. It shows the three tables where we want to map between. All of the three tables are located in the master zone inside the Data Lake.

Figure 5 shows three tables. The string_proteins table contains the information from the STRING database (ensembl ID, taxonomy ID and the pubmed ID of the article); the mapping table contains the mapping between the different type of protein identifiers from different sources (ensembl ( https://www.ensembl.org/info/genome/stable_ids/index.html) and uniprot ( https://www.uniprot.org/help/accession_numbers); and the tissues table includes tissue information about the protein (uniprot identifier, tissue identifier and tissue name) ( Figure 5).



**SELECT** string_proteins.ens_id,
**FROM** master.string_proteins
**LEFT JOIN** master.mapping **ON** string_proteins.ens_id =mapping.ens_id
**LEFT JOIN** master.tissues **ON** mapping.uniprot_id = tissues.uniprot_id
**WHERE** tissues.bto_name = ‘brain’;

The SQL query will connect the three tables through the matching protein identifiers, selecting only those which are expressed in the brain according to the matching identifiers (light green and purple). The query results in the first two proteins from the string_proteins table (light green color). If the script does not find any match between the ensembl and uniprot IDs, it skips them, like in the case of the third protein in the string_proteins table.

### Use Case 2: Tissue specificity

In this example, we would like to query the top 100 most highly expressed genes in a given tissue (in this case the human colon), and their protein interactors. The limitation with this use case is similar to the previous one: the user has to download the different structured interaction databases from web resources and write scripts or use online tools to work with the data at once. These are also prerequisite steps with the Sherlock platform (having to download the different sources and running preprocessing steps before working with them), but with the provided loader scripts, the user only has to do it once, and it is less time consuming. With Sherlock, the user can easily get this data from multiple interaction tables at once very quickly, thanks to the ORC format with the following SQL query:



**SELECT** molecule_id, molecule_id_type, tissue_uberon_id,tissue_uberon_name, score, interactor_a_id, interactor_b_id

**FROM** master.bgee_2020_11_16 bgee
**LEFT JOIN** master.omnipath_2020_10_04 **ON** bgee.molecule_id =omnipath_2020_10_04.interactor_a_id

**LEFT JOIN** master.omnipath_2020_10_04 **ON** bgee.molecule_id =omnipath_2020_10_04.interactor_b_id

**WHERE** tax_id = 9606
**AND** tissue_uberon_id = 1155
**ORDER BY** score **DESC**

**LIMIT** 100;


This query will select the top 100 highly expressed genes from a table imported from the BGee resource ( Bastian et al. 2021) to the master zone. The result will be filtered to return only those genes, which are expressed in the colon, and the query will select their protein interactors as well. The results are ordered by the score, so only the colon genes with the top 100 score and their first neighbours will return as the results.

### Use Case 3: Network enrichment

**Figure 6.**
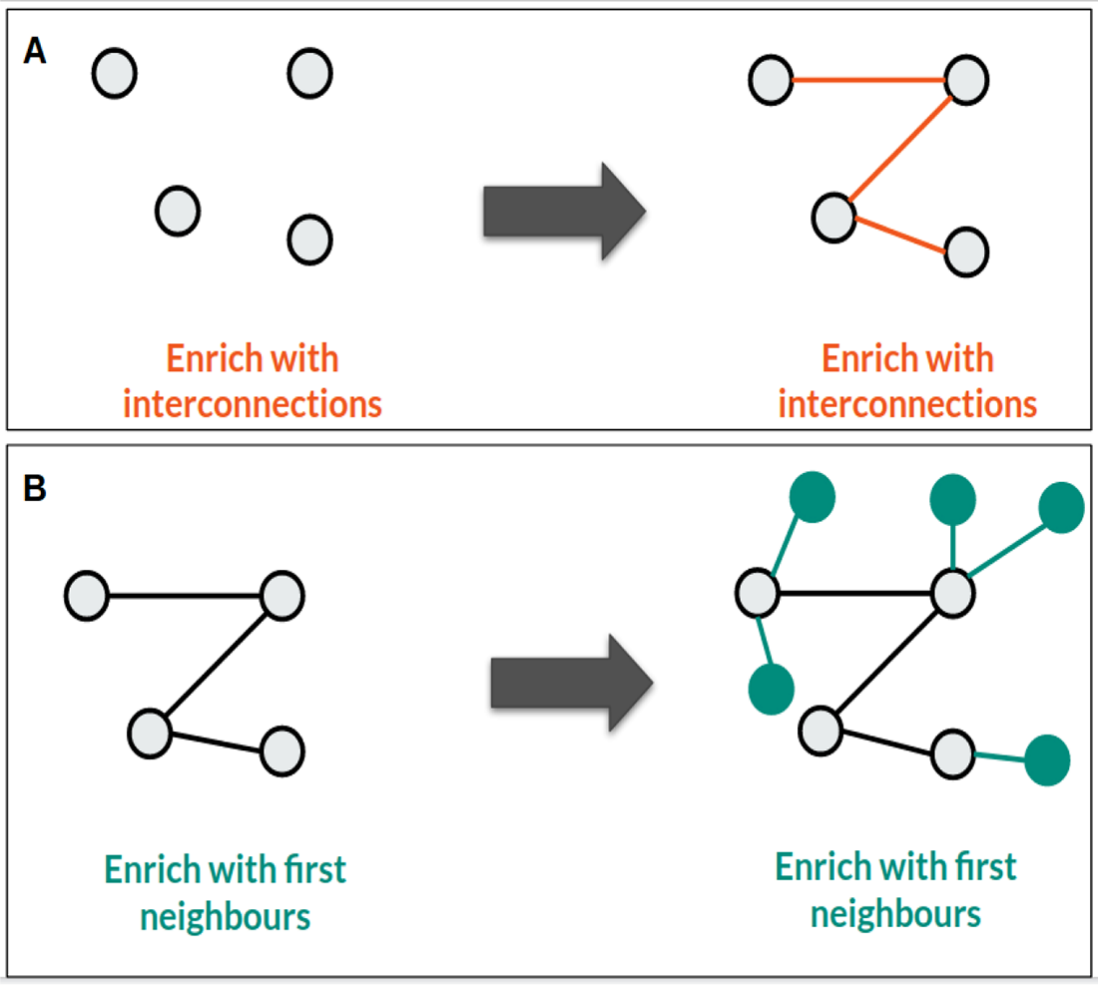
Example query, how can we map between different identifiers with Sherlock. **A**: Here you can see how we can find interconnections between our four chosen proteins. **B**: How can we enrich our network with the first neighbours of the given proteins.

In the third example, we would like to enrich a network with interaction data from the Data Lake with the help of Sherlock. We have certain proteins of interest shown on Figure 6, and we would like to investigate if there is any relationship between them (find the interactions). We would also like to enrich this network and our proteins with their first neighbours, using an interaction table loaded from the OmniPath ( Türei et al.) ( https://omnipathdb.org) database.

The SQL query for finding the connections between the proteins ( Figure 6A) is the following:



**SELECT** interactor_a_id, interactor_b_id

**FROM** master.omnipath_2020_10_04

**WHERE** interactor_a_id **IN** ('o95786', 'q96eq8', 'q6zsz5','q01113')
**AND** interactor_b_id **IN** ('o95786', 'q96eq8', 'q6zsz5','q01113');



To enrich our network with the first neighbours of the given proteins we have to use a different SQL query ( Figure 6B), as below:



**SELECT** interactor_a_id, interactor_b_id

**FROM** master.omnipath_2020_10_04

**WHERE** interactor_a_id **IN** ('o95786', 'q96eq8', 'q6zsz5','q01113')
**OR** interactor_b_id **IN** ('o95786', 'q96eq8', 'q6zsz5','q01113');



Only a single logical operator changed between the two queries (AND > OR). The reason is, if we want to find the interactions between the proteins, the query has to select only those interactions from the OmniPath table, where the source and the target protein are uniformly included among the proteins we examined. But in the other case, when we want to enrich the network, it is enough to select all of the interactions where either the source or the target proteins are among the proteins of our interest.

## Discussion

Sherlock provides a new, gap-filling method for computational biologists, who want not only to store, but very easily and quickly convert, query, generate or even share biological data. This novel platform provides a simple, user-friendly interface to work with common and widely used big data technologies, such as Docker or PrestoDB. Thanks to the ORC format that Sherlock uses, users can work with extremely large datasets in a relatively short time that can facilitate any given research project.

Since we made Sherlock, plenty of platforms like Sherlock have appeared worldwide, but none of them was designed specifically to the field of biology. One of them for example is the Qubole platform ( https://www.qubole.com). This platform also offers data storage and analytical queries solutions as well, but the Sherlock platform is cheaper. The data storage place and the virtual machines are also needed to be paid, but all of the source code is freely available and can be customized to the user's liking. On the other hand, Sherlock is specifically designed for biologists. It contains specific database loader scripts which they are able to create and upload the specific file formats to the Data Lake. We are constantly upgrading the already included datasets in our Data Lake. We will provide in the Github repository new database loader scripts (to extend interaction and expression data) and different mapping scripts as well. We will also develop different converting scripts to easily handle between Sherlock compatible and other file formats, such as TSV (tab separated value) or CSV (comma separated value).

Sherlock has a lot of features, which are the followings: 1) store all datasets in redundant and organized cloud storage, 2) convert all datasets to common, optimized file formats, 3) execute analytical queries on top of data files, 4) share datasets among different teams/projects, 5) generate operational datasets for certain services or collaborators, 6) it is really useful for any groups/teams in the field of computational biology, who has to work with very large datasets for their projects.

In the future, we would like to include and upload more and more database loader scripts to cover as many interaction and expression databases as possible. Furthermore we are planning to improve our source code, to make more detailed documentation. Right now, the update of the already included databases in the Data Lake is done manually, but we also would like to automate this with a script, which can handle all of the updates at once. Furthermore, we would like to include more common and general – in the field of biology – examples and show it in the repository. Furthermore, we are planning to develop some tutorials on how Sherlock can be used easily and what information is needed for the given projects. We are going to create short online courses and tutorials as well. Our main goal is to disseminate the Sherlock platform widely and we hope it can be useful and great help to the researchers.

## Conclusion

Sherlock provides an open-source platform empowering data management, data analytics and collaboration through modern big data technologies. Utilizing the dockerization of Presto and the Hive Metastore, Sherlock is not only powerful but also a flexible and fast solution to effectively and efficiently store and analyze large biological datasets. Sherlock can be used to execute queries on top of a Data Lake, where all the data is stored in a ‘folder-like’ structure providing the added benefit of well defined folder structures also helps and encourages correct data management of biological data. Having a scalable query engine and using ORC format, Sherlock can run SQL queries much faster than other solutions, meaning the user can spend more time working with the data than waiting for a search result, which can significantly reduce the lead time of the research project. In conclusion, Sherlock, through the repurposing concepts and open source tools created by large software companies, provides a ‘plug and play’ state-of-the-art data storage platform for large biological datasets.

## Data availability

All data underlying the results are available as part of the article and no additional source data are required.

## Software availability

Software available from: https://earlham-sherlock.github.io/

Source code available from: https://github.com/earlham-sherlock/earlham-sherlock.github.io

Archived source code available from: http://doi.org/10.5281/zenodo.4738516 ( Bohár et al. 2021)

License: MIT
