# Sherlock: an open-source data platform to store, analyze and integrate Big Data for computational biologists

**DOI:** 10.12688/f1000research.52791.2

**Published:** 2022-08-10

**Authors:** Balazs Bohar, David Fazekas, Matthew Madgwick, Luca Csabai, Marton Olbei, Tamás Korcsmáros, Mate Szalay-Beko

**Affiliations:** 1 Earlham Institute, Norwich Research Park, Norwich, UK; 2 Department of Genetics, Eotvos Lorand University, Budapest, Hungary; 3 Gut Microbes and Health Programme, Quadram Institute Bioscience, Norwich Research Park, Norwich, UK; 4 Department of Metabolism, Digestion and Reproduction, Imperial College London, London, UK

**Keywords:** Software, Computational biology, Network biology, Systems biology, Data lake, big data, data management, data integration

## Abstract

In the era of Big Data, data collection underpins biological research more than ever before. In many cases, this can be as time-consuming as the analysis itself. It requires downloading multiple public databases with various data structures, and in general, spending days preparing the data before answering any biological questions. Here, we introduce Sherlock, an open-source, cloud-based big data platform ( https://earlham-sherlock.github.io/) to solve this problem. Sherlock provides a gap-filling way for computational biologists to store, convert, query, share and generate biology data while ultimately streamlining bioinformatics data management. The Sherlock platform offers a simple interface to leverage big data technologies, such as Docker and PrestoDB. Sherlock is designed to enable users to analyze, process, query and extract information from extremely complex and large data sets. Furthermore, Sherlock can handle different structured data (interaction, localization, or genomic sequence) from several sources and convert them to a common optimized storage format, for example, the Optimized Row Columnar (ORC). This format facilitates Sherlock’s ability to quickly and efficiently execute distributed analytical queries on extremely large data files and share datasets between teams. The Sherlock platform is freely available on GitHub, and contains specific loader scripts for structured data sources of genomics, interaction and expression databases. With these loader scripts, users can easily and quickly create and work with specific file formats, such as JavaScript Object Notation (JSON) or ORC. For computational biology and large-scale bioinformatics projects, Sherlock provides an open-source platform empowering data management, analytics, integration and collaboration through modern big data technologies.

## Introduction

Most bioinformatics projects start with gathering a lot of data. As bioinformaticians work on bespoke or public datasets (for example, gene expression or mutation datasets), they require external reference data in almost all cases. Most projects may call for genome annotations, gene ontologies, tissue-specific expression datasets, drug-related databases, and many other reference datasets. One of the reasons why we use the term ’bioinformatics’ in the first place is because we cannot correlate and process all these datasets manually. We need the help and the power of computers and databases ( Greene *et al.*, 2014). Thanks to the current technical advancement, many solutions exist worldwide to utilize the available computational possibilities ( Marx, 2013).

Cloud storage solutions such as Amazon’s S3 ( https://aws.amazon.com/s3/) or Google Cloud Storage ( https://cloud.google.com/storage) offer scalability and flexibility to the matching compute solutions. More importantly, they allow the storage of large datasets on the same infrastructure as the large-scale analyses, with the additional benefit of allowing data sharing across teams and collaborators. Some companies have utilized cloud resources to offer storage, access to shared datasets, and transparent data sharing. Cloud storage also improves reliability, as the data is backed up in several geographical locations ( Kasson, 2013). However, in most of the cases, these cloud storage companies only offer data storage solutions, but this does not include platforms to execute or work with data.

To address these issues, we repurposed concepts and open source tools from the top players of the software industry, like Facebook (Meta), Netflix and Amazon. To replace the tedious process of manual data collection before the first steps of any bioinformatics project, we developed a new platform, Sherlock.

The Sherlock platform has two main parts:

- a query engine, which is responsible for executing the given Structured Query Language (SQL) queries
- a Data Lake, required for data storage, which consists of multiple different databases or datasets, where the Query engine can execute queries against the data. The combination of these two parts in Sherlock streamlines efficient data collection, data integration, and data preprocessing ( Khine & Wang, 2018).

## Implementation and operation

### Overview of the Sherlock platform

**Figure 1.**
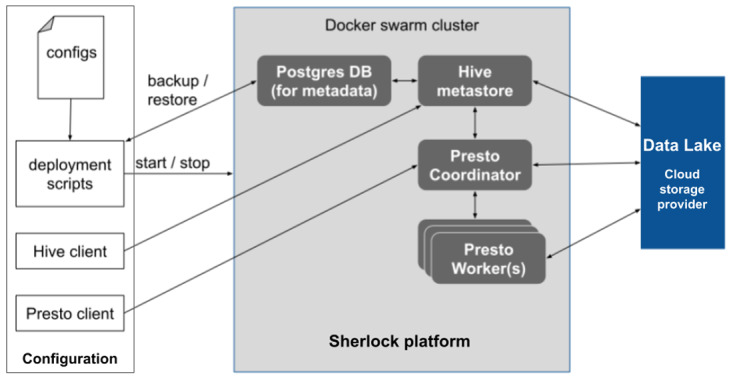
Overview of the Sherlock platform and its relationship with the Data Lake. The schematic shows how the different tools and the deployment modules interact inside the core of Sherlock. Only the Hive Metastore, the Presto Query Engine and the possible worker nodes have a connection to the Data Lake. The blue box represents the Data Lake, where the data is stored.

Sherlock is an open source and freely accessible platform that combines several different software technologies ( https://earlham-sherlock.github.io/). One core software that Sherlock uses is Docker and the Docker swarm. It is essential to clarify that Sherlock is only a platform, which means it does not include data; instead, it provides a dockerized platform where one can work on and manipulate the data ( Figure 1). Then by leveraging robust database architectures (Presto query engine and Hive metastore) and providing them with a channel to connect to the Data Lake (where the data is stored), the user can run different analytical queries on top of the data files.

As described in the introduction, Sherlock is flexible and can handle bespoke or public data. On the one hand, it can convert any bespoke structured data in a table into a standard file format used by a platform. On the other hand, with the scripts in the GitHub repository ( https://github.com/earlham-sherlock/earlham-sherlock.github.io/tree/master/loaders), Sherlock can work with different publicly available databases as well.

### Architecture

***Docker & Docker Swarm*.** Docker is an open and widely used technology to isolate Linux processes and provide them with a reproducible, well-defined runtime environment independent of the operating system ( Matthias & Kane, 2015). Each element in the Sherlock platform is running inside a separate Docker container. A Docker container is a standard unit of software that packages up all of the necessary code and all the dependencies, so the application runs quickly and reliably in an isolated environment on any machine. Container orchestration tools are used when a complex solution (like Sherlock) requires the interconnection of many docker containers distributed among multiple separate Linux machines. In Sherlock, we are using the standard Docker Swarm orchestration platform ( Smith, 2017), as it is easier to use and operate than other orchestration frameworks (like Kubernetes ( https://kubernetes.io)). These industry-standard technologies make Sherlock's management (starting, stopping and monitoring) straightforward while enabling us to implement more advanced features, for example, the live on-demand scaling of the Sherlock’s analytical cluster. This scalability is the most significant advantage of Docker Swarm. The entire cluster can be shut down or reduced to the minimum requirements in the cloud when it is not used. Then, it can be scaled up to hundreds of nodes if necessary for executing very complex analytical queries. Sherlock uses a hierarchical structure to achieve this, where several worker nodes and at least one manager node are responsible for handling the worker nodes' resources and ensuring that the cluster operates efficiently.

**Figure 2.**
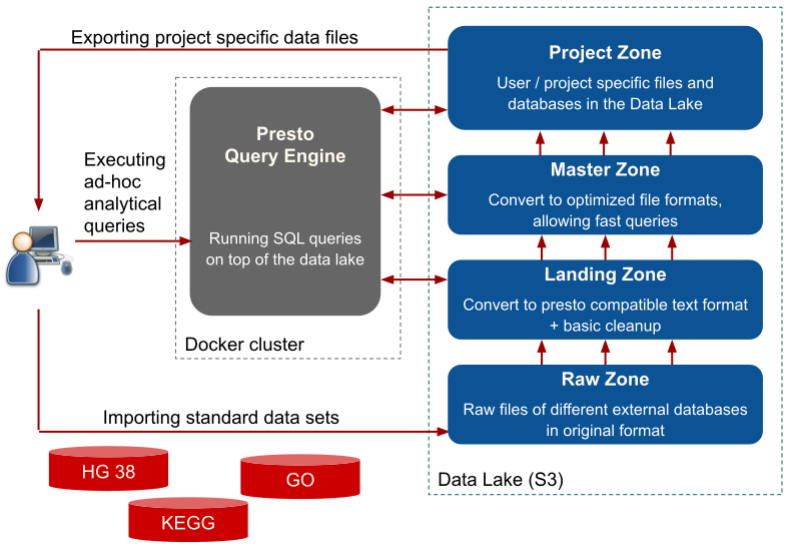
schematic representation of the relationship between Sherlock's two main components: the Query Engine (dockerized) and the Data Lake. **Left side:** The core component of the platform: the Presto Query Engine. It allows the user to execute different analytical SQL queries on top of the data files in the Data Lake (right side). **Right side:** The structure of our Data Lake. They are separated into four different zones. Working from bottom to top; 1) The Raw Zone contains the raw data from the different external or bespoke databases in their original formats. 2) The landing Zone, where the data is in JSON Lines format, is compatible with the Presto Query Engine. 3) The master zone, where the data is in a common optimized and compressed format, is called ORC. This format enables faster query execution than the landing zone. 4) The Project Zone, where we store exclusively the data needed for specific/active projects.

***Query Engine*.** The first core part of Sherlock is the Query Engine ( Figure 2), which is responsible for running the given question through SQL commands on top of the data files and retrieving the ‘answer’ for the user. In general, query engines (Hive, Impala, Presto) “sit” on top of a database or server and execute queries against data. More specifically, many of them use SQL query engines for C-R-U-D (create, read, update, delete) operations. Query engines facilitate access to the data in relational systems and enforce data policies that relational data models and database management systems require. In Sherlock, we are using the Presto Query Engine ( https://prestodb.io), developed by Facebook. Presto provides a high-performance Query Engine wrapped in an easily deployable and maintainable package. Furthermore, the Presto Query Engine can handle many different types of data storage solutions.

With Presto, we can formalize analytical questions using SQL. SQL can be used for stream processing in a relational data stream management system (RDSMS). Moreover, one of SQL's most beneficial features is handling structured data, such as data that resides in a fixed field within a table, allowing us to easily search and manage the data. The data can be arranged and analyzed in various ways, such as sorting alphabetically or totalling a set of values ( Silva *et al.*, 2016). When combined with a Query Engine, SQL can be used to connect to the Data Lake, read and integrate the data stored within it and execute different analytical queries, either sequentially or in parallel, depending on the power of the given machine.

***Hive Metastore*.** The second core part of Sherlock is the Hive metastore ( https://docs.cloudera.com/runtime/7.0.1/hive-metastore/topics/hive-hms-introduction.html), which is a service that stores metadata related to Apache Hive and other services, in a backend RDBMS, such as MySQL or PostgreSQL. In Sherlock, the Hive metastore contains only the meta-information about the different tables (their schema or location) in the Data Lake for the Presto Query Engine to query. The Hive metastore is the intermediate layer between the Presto query engine and the data lake, enabling Presto to access the meta-information and query the desired data. Sherlock provides simple scripts to save this metadata in the Data Lake, either when the user wants to make a backup or before the termination of the analytical cluster.

### Deployment

For Sherlock, we developed a dockerized version of Presto that also incorporates the Hive metastore. This addition of the query engine and metastore makes Sherlock cloud-agnostic, so it can be installed at any cloud provider, providing it in a Linux machine with Docker installed. The advantage of running Presto in a dockerized environment is that it enables the automatic installation and configuration of the whole Presto Query Engine. Furthermore, the whole platform can be fired up on a local machine, multiple machines or any cloud service. We show how to make a set of Linux virtual machines with Docker installed, then start a distributed Presto cluster by using the Sherlock platform under the deployment section of the GitHub page of Sherlock ( https://earlham-sherlock.github.io/docs/deployment_guide.html).

### The Data Lake – data storage

A Data Lake is a simple network storage repository that can be accessed by external machines ( https://aws.amazon.com/big-data/datalakes-and-analytics/what-is-a-data-lake/). All the data imported into, transformed in, or exported from Sherlock will be stored here as simple data files. The most significant advantage of using a Data Lake is that its operation will be identical on a local machine as on the cloud. Therefore, the data stored in the Data Lake is well-structured and placed in reliable storage. Furthermore, the Data Lake can be scaled independently from the query engine. The technologies in Sherlock are compatible with both most common Data Lake solutions, Hadoop Distributed File System (HDFS), a distributed file system designed to run on commodity hardware, and the Simple Storage Service (S3) storage formats. However, we only described S3 in all of our examples, as S3 is more widely-used, modern, and accessible. Although HDFS is an older standard solution, it has some compelling features which can result in better performance. Still, it is much more challenging to set up, maintain and in most cases, its extra features are not necessary for the given project. In contrast, S3 is a standard remote storage API (Application Programming Interface) format, introduced first by Amazon ( https://aws.amazon.com/s3/). As of writing, users can purchase S3 storage as a service from all major cloud providers like Digital Ocean, Amazon AWS, Google Cloud or Microsoft Azure. Each of these can be compatible with the Sherlock platform.

Having somewhere to store the data is only half of having an operational Data Lake. One can not stress enough how important it is to organize the data in a well-defined ‘folder’ structure. Many Data Lake deployments become unusable after a few years due to poor maintenance, resulting in a large amount of data ‘lost’ in the thousands of folders or inconsistent file formats and no ownership over the data. Our solution is to separate all of the data in the Data Lake into four different main folders, representing different stages of the data and different access patterns in the Data Lake. Inside each of these main folders, we can create subfolders, and it is a good practice to incorporate the name of the dataset, the data owner name, the creation date (or other version info), and the file formats somehow into their paths.

**Figure 3.**
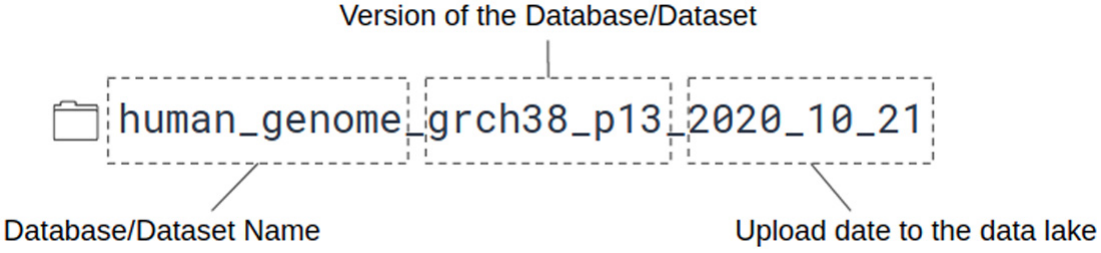
An example naming convention for data stored inside the Data Lake. The first part of the folder name is the name of the given database/dataset. In this example case, the human genome. The next part is the version of the data, and the last is the uploaded date to the Data Lake. https://jsonlines.org/ https://orc.apache.org/docs/

We separated our Data Lake into four main zones, which are built on top of each other ( Figure 2). The first zone is the raw zone. We archived all the database files into the raw zone in their original formats. For example: if we downloaded the human genome, then we put the fasta files here, under a separate subfolder. The subfolder’s name should contain the exact version (for example: hg38_p12) ( Figure 3), and also, we put a small readme file in the folder, where we listed some metadata, like the date and the URL of the download, etc. Usually, these files cannot be opened with Presto, as the file format is incompatible in most cases.

The next zone is the landing zone. We needed to develop specific loader scripts, which convert and extract the raw datasets into the landing zone. We converted the data to a text file in JSON Lines format ( https://jsonlines.org), which Presto can open. JSON Lines is a specific JSON format, where each text file line represents a single JSON record. Each JSON file for a given dataset is placed into a separate sub-folder in the landing zone. It is then registered by Presto which sees the dataset now as a table. Then, Presto will be able to load the data and perform other operations, for example it can execute simple data transformations. However, we do not recommend using the tables in this zone for querying because processing the large JSON files is very slow.

Using Presto, we converted the data from the landing zone into an optimized (ordered, indexed, binary) format for the next zone, which is the master zone. The main idea is that we use the tables in the master zone later for analytical queries. Here the data is in a more detailed and exact format, called Optimized Row Columnar (ORC), which is a free and open-source column-oriented data storage format ( https://orc.apache.org/docs/). With ORC, Sherlock can perform SQL queries much faster than the JSON text file format from the previous zone (landing zone). If necessary, advanced bucketing or partitioning on these tables can be used to optimize the given queries. Furthermore, the master zone contains the ‘single source of truth’. This means that the data here cannot be changed, only extended upon, for example, adding a new version of the datasets.

**Figure 4.**
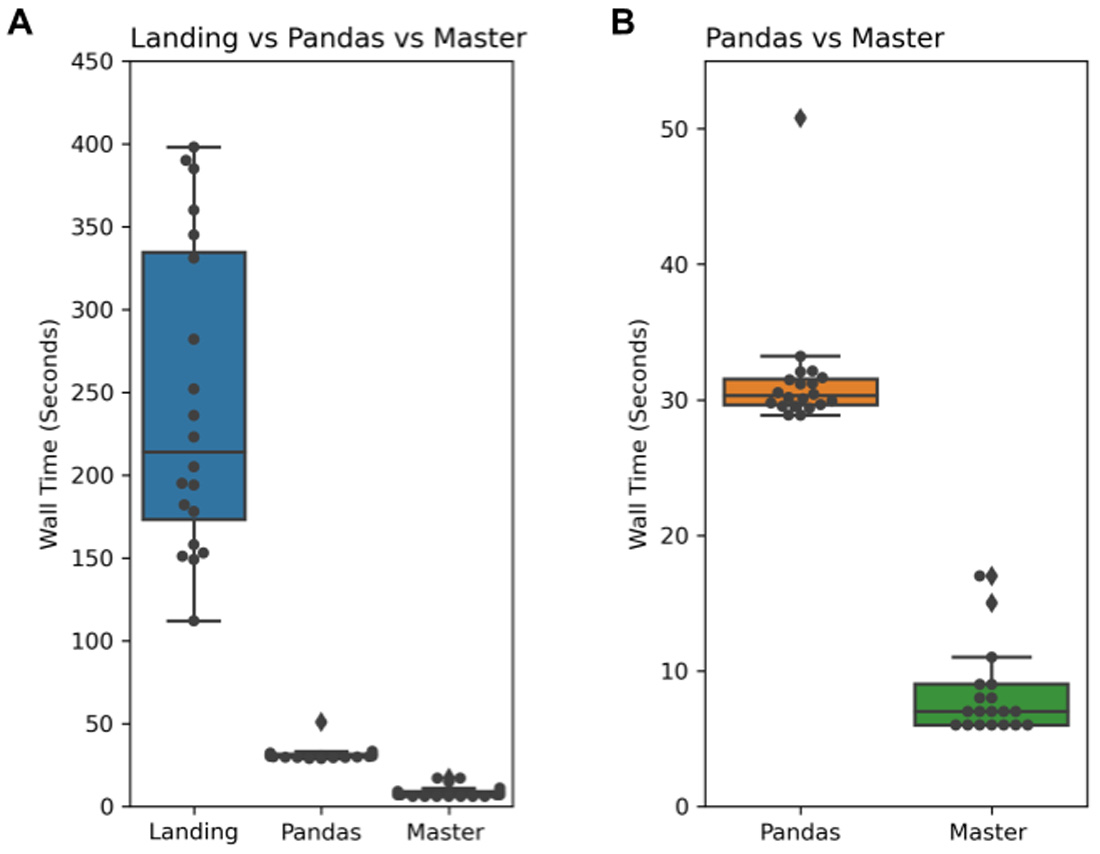
Results of the average run time (in seconds) of executing 20 queries on the landing zone vs Pandas vs the master zone. **A**: The wall time comparison between the three different approaches for 20 repeated queries. After evaluating the run time performance, queries running in the master zone are ~20–30 times faster than those in the landing zone and ~3–4 times faster than Pandas. The significant deviation in the landing zone is due to the connection with S3 on Digital Ocean. During longer queries, it can result in a bottleneck hence the need for master zone's optimized data format. **B**: The speed difference between Pandas and the master zone’s ORC format. The master zone queries ~3–4 times quicker than Pandas. Therefore, it demonstrates the performance increase of adopting the ORC format in the master zone, which enables this increase in performance.

For illustrating the query time speed difference between the landing and the master zone, we ran the following SQL query in both zones 20 times ( Figure 4):


SELECT bgee.molecule_id, uni.to_id bgee.molecule_id_type, bgee.tissue_uberon_id, bgee.tissue_uberon_name, bgee.score

FROM bgee_2021_04_05 bgee

LEFT JOIN uniprot_id_mapping_2021_04 uni

ON uni.from_id_type = 'ensembl_gene_id'

AND uni.to_id_type = 'uniprotac'

AND uni.from_id = bgee.molecule_id

WHERE bgee.tax_id = 9606

AND bgee.tissue_uberon_id = 2113

AND uni.to_id IS NOT NULL

ORDER BY score DESC

LIMIT 100;


This query is selecting the top 100 most highly expressed genes in a given tissue (the human liver) and their UniProt identifiers. In both zones, for this query, we used the UniProt ( UniProt Consortium, 2021) mapping table (version 2021_04 from the UniProt website) and the Bgee ( Bastian *et al.*, 2021) table (downloaded from the Bgee database on 5th April 2021). Both tables have a large file size and number of rows: the UniProt mapping table is 1.1 GB and it has 25,458,069 and the Bgee table is 550 MB and it has 2,876,219 rows. Despite this huge amount of data in each table, the queries running in the master zone are 20–30 times faster than the queries in the landing zone, because of the ORC format.

Additionally, we benchmarked the master zone and Pandas, an industry-standard python package used for data wrangling ( https://pandas.pydata.org), to compare their performance. For this comparison, we converted the landing zone tables into tab-separated value (TSV) format and generated the same query as seen above in Pandas. We repeated this experiment 20 times to select the human liver's top 100 most highly expressed genes as outlined in the query above. We found that Sherlock is still 3–4 times quicker than pandas at performing the example query ( Figure 4).

The last zone is the Project zone. This zone saves and holds the tables needed only for specific projects. We can even create multiple project zones, for each group, project or user. However, it is crucial to have a rule implemented to indicate the owner of the tables; as mentioned before, this dramatically increases the effectiveness of Sherlock and ease of maintenance.

The main reason for having this multi-level folder structure is because of reusability. With the raw and processed data in the different zones, the users can easily access all the data and there is no need to spend unnecessary time acquiring them again. The other part is that the data is more specific and compressed in the upper zones, allowing for running fast and efficient SQL queries. The other advantage is that storage space is cheaper compared to computing capacity. In light of this, if we store our data in several copies, we do not have to pay significantly more. This allows us to keep the raw data and increase the chance of reusability.

### Functionality of Sherlock

**Figure 5.**
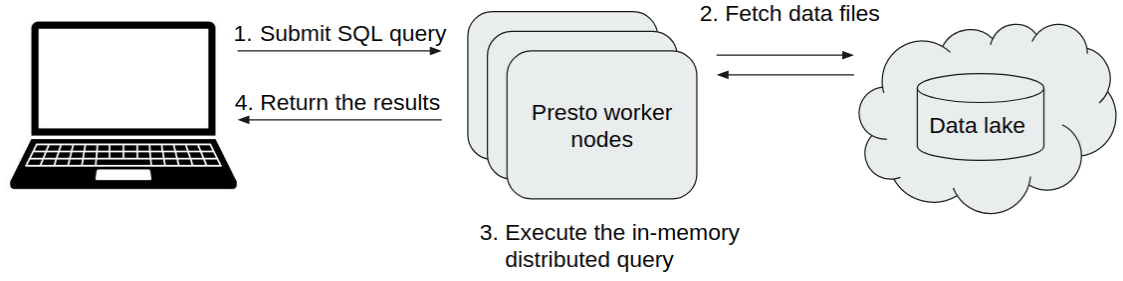
Overview of how the query engine and the Data Lake work together. This schematic represents the different steps, how a user can submit an analytical SQL query and a summary of the workflow of this query until the results are returned to the user.

Here, we describe the key steps of how the query engine and the Data Lake can be used together ( Figure 5). The first step is to set up the platform itself by starting a Docker Swarm with the deployment modules on a cluster with several worker nodes. These modules start different services, but each in a separate container. These deployment modules are configurable ( Figure 1). One can specify exactly how many resources a service can use simultaneously (e.g. CPU and memory allocation). Inside a running Docker Swarm, Sherlock has two main parts: the Hive Metastore and the Presto query engine. Sherlock follows the industry best practices in its architecture to enable scalability. Usually, the main idea behind scalable batch processing architectures is to separate data storage and data analytics ( Dean & Ghemawat, 2008). This architecture allows us to scale the analytical power and the storage size independently and even dynamically. This is the case with Sherlock when deployed in the cloud. The basic order of operations is as follows; an SQL query is submitted to the Presto query engine, then it will connect to the Data Lake and fetch the necessary data files. Presto executes the in-memory distributed query and then it returns the results to the user ( Figure 5).

Sherlock has been designed for computational biologists, especially for network and systems biologists. It can contain specific interaction, expression and genome data-related databases with the help of the loader scripts that can create the specific file formats from the source databases and upload them into the Data Lake. With these individual loader scripts, users can make and work with the necessary file formats from the different data sources without having to develop their own loading scripts. The whole source code of the platform and the loader scripts ( Table 1) are freely available on GitHub: https://github.com/earlham-sherlock/earlham-sherlock.github.io

**Table 1.** The specific loader scripts, which are already included in Sherlock’s GitHub repository.

Datatype	Loader script	Source	Reference
**General datasets**	DBSnp database	https://www.ncbi.nlm.nih.gov/snp/	( Smigielski *et al.*, 2000)
	Gene Ontology	http://geneontology.org	( Ashburner *et al.*, 2000)
	Gene Ontology Annotations		
	Human Genome	https://www.gencodegenes.org	-
	Uberon Gene Ontology	https://www.ebi.ac.uk/ols/ontologies/uberon	( Mungall *et al.*, 2012)
	Uniprot ID Mapping data	https://www.uniprot.org	( UniProt Consortium, 2021)
**Interaction ** **databases**	BioPlex database	https://bioplex.hms.harvard.edu	( Huttlin *et al.*, 2020)
	Dorothea database	https://dorothea.opentargets.io/#/	( Garcia-Alonso *et al.*, 2018)
	HINT database	http://hint.yulab.org	( Das & Yu, 2012)
	HuRI database	http://www.interactome-atlas.org	( Luck *et al.*, 2020)
	InBioMap database	https://inbio-discover.com	( Li *et al.*, 2017)
	IntAct database	https://www.ebi.ac.uk/intact/	( Orchard *et al.*, 2014)
	IRefIndex database	http://irefindex.uio.no	( Razick *et al.*, 2008)
	Mentha database	https://mentha.uniroma2.it	( Calderone *et al.*, 2013)
	Omnipath database	https://omnipathdb.org	( Türei *et al.*, 2021)
	STRING database	https://string-db.org	( Szklarczyk *et al.*, 2019)
**Expression data**	Bgee database	https://bgee.org	( Bastian *et al.*, 2021)

It cannot be emphasized enough that maintaining the data and a well-defined folder structure in the Data Lake is essential. Furthermore, keeping the folders and the tables in the Data Lake clean and very documented is vital. Often a user may not have been the one who uploaded or generated the data, which sometimes makes it difficult to gain an understanding of what a table contains. Without this knowledge, a user would struggle to formulate a query. There are two straightforward solutions to this. We provide a Web User Interface for Sherlock where a user can check the Schemas of tables held in the Data Lake. Alternatively, a more technical one, where the user can check the columns' names using a simple SQL query. From our experience, however, the best practice to check the content of the different tables is to create a wiki or a well-documented description of the master (query) zone, where everybody can find the needed information and the column structure of the different tables.

In the next section, we will outline three different use cases of Sherlock, describing how to set up the Sherlock platform and how to load data into its Data Lake.

## Use cases

### Platform and Data Lake set up

**Figure 6.**
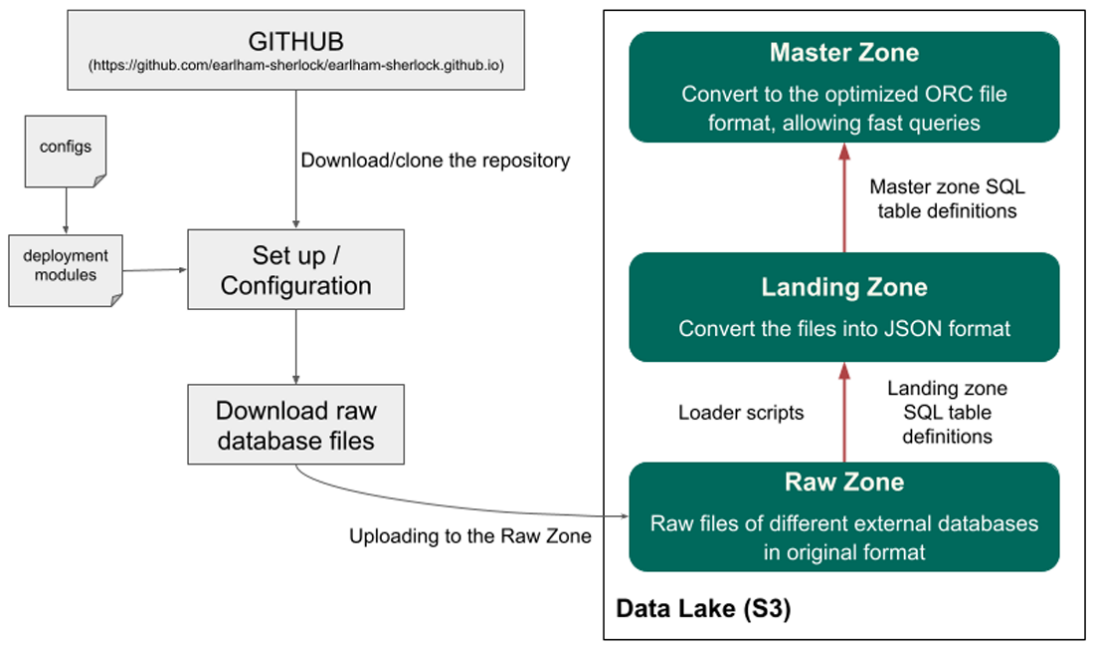
This figure represents the different steps how the whole platform can be configured and how data can be uploaded to the Data Lake.

Before we introduce some relatively simple use cases of how the Sherlock platform can be used with a Data Lake, we will first explain the fundamentals of the platform. This consists of downloading the GitHub repository, loading data to the Data Lake with the help of the different loader scripts and using the SQL language to execute analytical queries ( Figure 6).

As described in the previous section, the first step of using the platform is to set it up by downloading the GitHub repository ( https://github.com/earlham-sherlock/earlham-sherlock.github.io). Within the repository, extensive documentation and a readme file describe how to commission the platform on various solutions. This includes on a single laptop/computer or a cluster hosted by a cloud provider. After the successful operational set-up step, data can now be uploaded to the Data Lake. First, we have to download the whole database of interest and upload them to the raw zone of the Data Lake. With the help of the loader scripts, we can convert the raw database files into JSON file formats, which can be uploaded and registered in the landing zone with PrestoDB using a simple SQL query. An example query can be found on the GitHub repository ( https://github.com/earlham-sherlock/earlham-sherlock.github.io/tree/master/table_definitions/landing_zone). This step allows the Hive Metastore to see the structure of the tables in the landing zone (columns of the tables). Because of this, we can query this zone as well, but as we highlighted before, it will be much slower than the master zone. With simple SQL queries, we can convert the JSON tables from the landing zone into the ORC format and upload them to the master zone. An example of this can be found on the GitHub repository ( https://github.com/earlham-sherlock/earlham-sherlock.github.io/tree/master/table_definitions/master_zone).

After this, with the help of the configuration and setup modules, we have an operational platform connected to our data-filled Data Lake using the raw databases/files of interest. From this stage, there are a couple of opportunities to use the platform:

1) download the command line interface of PrestoDB ( https://prestodb.io/docs/current/installation/cli.html) and in terminal connect to the query engine with a simple command:

./presto --server localhost:8089 --catalog sherlock

2) Sherlock provides a simple Web User Interface (Web UI) for the coordinator virtual machine. This interface is not part of the general Presto Query Engine, it is an open-source and freely available tool, which is running in a separate docker container alongside the Sherlock platform ( https://yanagishima.github.io/yanagishima/). This can be linked together by adding the bind address of the ssh command:

ssh {coordinator virtual node IP/name} -L 8090:localhost:8090

**Figure 7.**
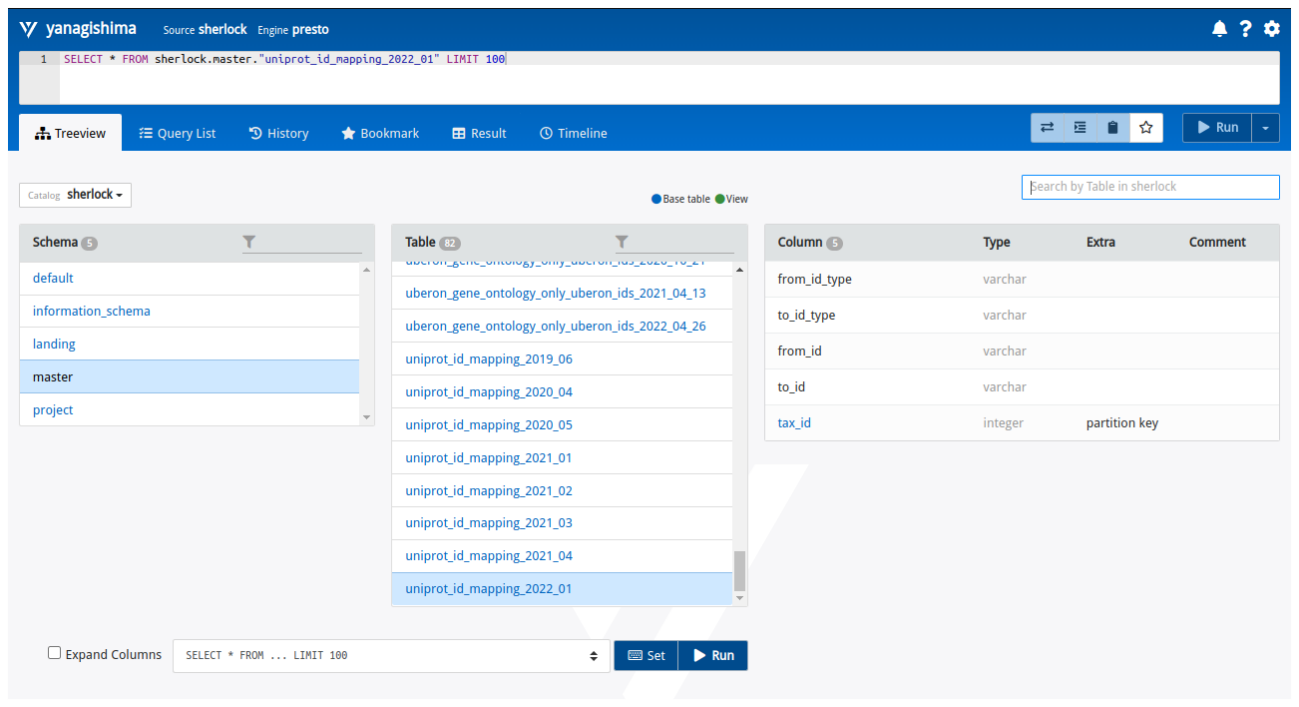
The Web User Interface (Web UI) for Sherlock. At the top, there is a command line interface where the user can write SQL queries manually. From left to right are the zones (schemas), the tables inside the zones, and the column structure of the table of interest.

Then opening a web browser and going to the localhost:8090 URL, the Web User Interface will come up ( Figure 7).

This Web UI has a simple user interface and some useful features that can help the users handle and manipulate the data inside the different zones without using a terminal. It has a built-in command line interface (CLI), where users can write their SQL queries. As we described earlier, it can give information about the contents of different tables (what the columns are) with the ‘Treeview’ menu point. The Web UI has a history feature that can help to search back the latest SQL queries and save the time to rewrite them. Furthermore, frequently used and essential queries can be bookmarked again to save the users time. Last but not least, after a user runs the given analytical SQL query or queries, they will get back the results and can download them in a table format.

In the following subsections, we will highlight three use cases of using the platform with our Data Lake, which contains the databases collected in Table 1.

### Use case 1: Identifier (ID) mapping

In this use case the goal is to show how Sherlock can run simple SQL queries with merging tables to map different identifiers. For this first use case, use the STRING table (downloaded on the 9th of September 2021) and the UniProt mapping table (which version 2021_03 from the UniProt website). Both tables were uploaded to the landing zone and then converted to the master zone using the steps described in the previous section. One of the most repetitive and crucial tasks for those working in bioinformatics is identifier (ID) mapping. Working with many different datasets from multiple sources, all carrying diverse identifiers, is challenging. In addition, these identifiers can have clashing structures, making it complicated and time-consuming to work with them - resulting in the development of different scripts or the use of various tools to work with them simultaneously. Users have to make different scripts or use various tools to work with these identifiers at once. Nowadays, the principal idea behind these ID mapping steps is to have a separate table or tables, called mapping tables, which contain the different identifiers. The best practice is to have only one mapping table, which includes all the necessary identifiers for a given project. The limitation of this approach is that when a team has to work with so many different identifiers at once, this mapping table can be large, which increases the computational time to extract the data from the mapping table. In Sherlock, users can work with just one mapping table, which can be made quickly with the help of the provided loader scripts from the GitHub repository, which can significantly shorten the time needed for ID mapping. Sherlock can execute these queries very quickly despite the large size of the mapping tables, owing to the implemented ORC format. To demonstrate this capability in Sherlock, we search for genes from the STRING table ( Szklarczyk *et al.*, 2019), and we would like to get the UniProt identifiers of these genes.

**Figure 8.**
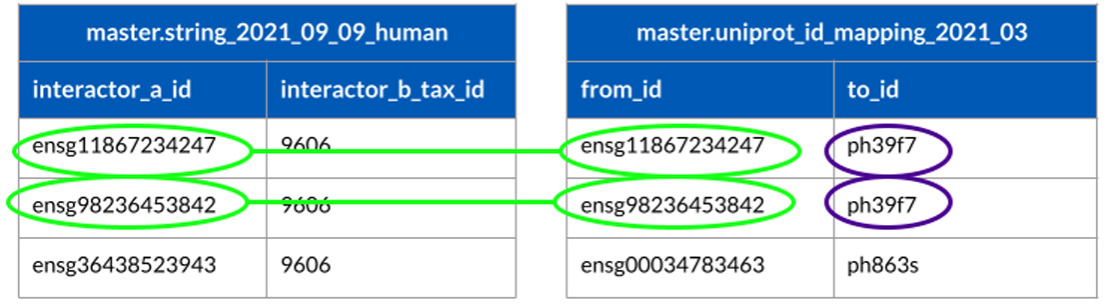
This schematics describing the mapping between identifiers of two tables in the master zone (‘string_2021_09_09_human’, which contains the human interactions from the STRING database and `uniprot_id_mapping_2021_03`, which contains the mapping information between different types of identifiers).

Two different datasets are needed to execute this example SQL query. Table one contains the information from the STRING database (not showing all of the columns from the actual table) and the second table is a mapping table containing the mapping between the different types of protein identifiers from different sources (Ensembl ( https://www.ensembl.org/info/genome/stable_ids/index.html) and UniProt ( https://www.uniprot.org/help/accession_numbers)) ( Figure 8). With the following example query, Sherlock can map between different identifiers, using two tables from the master zone.


SELECT to_id

FROM master.string_2021_09_09_human string

LEFT JOIN master.uniprot_id_mapping_2021_03 uniprot

ON string.interactor_a_id = uniprot.from_id


The SQL query will connect the two tables through matching protein identifiers, selecting only those expressed in the brain according to the matching identifiers (light green and purple in Figure 8). The query results in the first two proteins from the master.string_2021_09_09_human table (light green color). If the query does not find any matches between the Ensembl and UniProt IDs, it skips them. This is demonstrated in Figure 8, where the third protein in master.string_2021_09_09_human table does not have a match in the uniprot_id_mapping_2021_03 table.

### Use case 2: Tissue specificity

In this case, we would like to query the top 100 most highly expressed genes in a given tissue (the human liver) and their protein interactors. The limitation of this use case is similar to the previous one: the user has to download the differently structured interaction databases from web resources and then write new scripts, or use online tools, to get the data into a workable format. These are also prerequisite steps with the Sherlock platform, e.g. having to download the different sources and run preprocessing steps before working with them. However, with the provided loader scripts, the user only has to do it once, which is less time-consuming. With Sherlock, the user can quickly get this data from multiple interaction tables at once very quickly, thanks to the ORC format. In this example, we are using the BGee table and the OmniPath table from the master zone of the Data Lake. The BGee table was downloaded on the 5th of April 2021 and the OmniPath table was downloaded on the 13th of September 2021. Both tables were created and formatted with the steps from the beginning of the User Cases section.

With the following SQL query Sherlock can give the answer for what are the top 100 most highly expressed genes in the human liver:


SELECT molecule_id, molecule_id_type, tissue_uberon_id, tissue_uberon_name, score, interactor_a_id, interactor_b_id

FROM master.bgee_2021_04_05 bgee

LEFT JOIN master.omnipath_2021_09_13

ON bgee.molecule_id = omnipath_2021_09_13.interactor_a_id

LEFT JOIN master.omnipath_2021_09_13

ON bgee.molecule_id = omnipath_2021_09_13.interactor_b_id

WHERE tax_id = 9606

AND bgee.tissue_uberon_id = 2113

ORDER BY score DESC

LIMIT 100;


This query will select the top 100 highly expressed genes from a table imported from the BGee resource ( Bastian *et al.*, 2021) held in the master zone. The result will be filtered to return only those genes expressed in the colon, and the query will select their protein interactors from the Omnipath interaction database resource. The results are ordered by the scores, so only the colon genes with the top 100 scores and their first neighbors will be returned.

ref3

### Use case 3: Network enrichment

**Figure 9.**
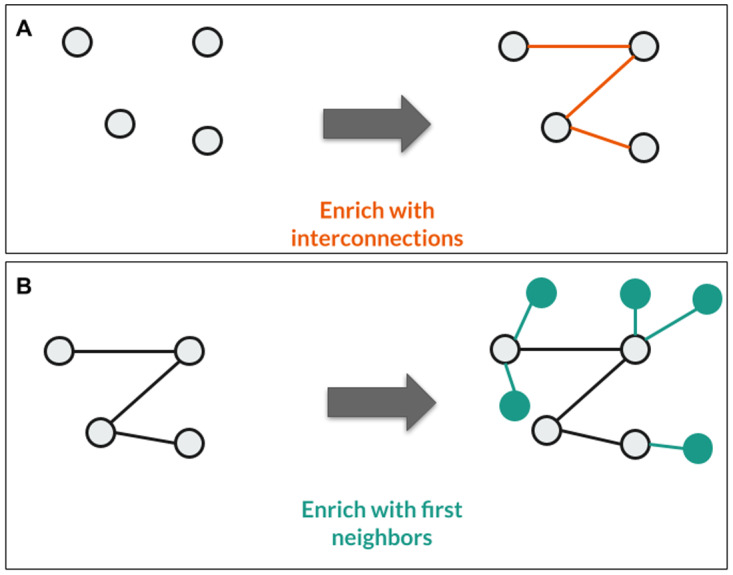
Representation of the third use case. **A**: With the given SQL query, Sherlock can find the interconnections between the selected proteins in this step. **B**: After a single logical operator change between the two queries (AND operator to the OR operator), Sherlock can also identify the selected proteins' first neighbors.

In the third example, the goal is to query the interconnections between specific genes of interest and their first interaction partners as well, using the OmniPath database from the Data Lake with the help of Sherlock ( Figure 9). It often happens in various bioinformatics projects that one needs the direct interaction partners of a given protein, or has to find the interconnections between different ones. With the help of the Sherlock platform one can query quickly on several different databases at the same time, which are in the Data Lake. This can definitely shorten the time required to answer the biological questions.

In this example, we only use a single interaction database, the OmniPath table from the master zone as in the second use case.

The SQL query for finding the connections between the proteins ( Figure 9, A) is the following:


SELECT interactor_a_id, interactor_b_id

FROM master.omnipath_2020_10_04

WHERE interactor_a_id

IN ('o95786', 'q96eq8', 'q6zsz5', 'q01113')

AND interactor_b_id

IN ('o95786', 'q96eq8', 'q6zsz5', 'q01113');


To find the first neighbors of the given proteins we have to use a different SQL query ( Figure 9, B), as below:


SELECT interactor_a_id, interactor_b_id

FROM master.omnipath_2020_10_04

WHERE interactor_a_id

IN ('o95786', 'q96eq8', 'q6zsz5', 'q01113')

OR interactor_b_id

IN ('o95786', 'q96eq8', 'q6zsz5', 'q01113');


Only a single logical operator changed between the two queries (AND in Figure 9. A, OR in Figure 9. B). The reasoning behind this is that if we want to find the interactions between the proteins, the query has to select only those interactions from the OmniPath table where the source and the target protein are uniformly included among the proteins we examined. However, in the other case, it is enough to select all interactions where the source or the target proteins are among the proteins of interest when we want to enrich the network.

## Discussion

Sherlock provides a new, gap-filling method for computational biologists to store, convert, query, generate or even share biological data efficiently and quickly. This novel platform provides a simple, user-friendly interface to work with every day and widely used big data technologies, such as Docker or PrestoDB. Sherlock leverages the ORC format so users can work with extremely large datasets in a relatively short computational time. Thus, Sherlock provides a platform to facilitate data management and analytics for biological research projects.

Some other existing platforms support Big Data in computational biology, for example, Galaxy ( https://galaxyproject.org) or AnVIL ( https://anvilproject.org). Galaxy and AnVIL are general workflow management platforms that provide workspaces. In these workspaces, a user can run workflows of analytical programs (tools), which were developed by someone else already. Public databases are often available through import tools in Galaxy, AnVIL or even through R. Sherlock on the other hand focuses more on storing and combining data sets and allowing flexible, ad hoc queries to manipulate this data or build new data sets based on the existing ones. Sherlock does not provide any workflow system; therefore, Sherlock cannot run custom code to perform complex analytical workflows (like running simulations on molecules or executing machine learning modules). Instead, it provides some basic data manipulation (filtering, joining, sorting) which enables to answer simple analytical questions. In brief, Sherlock focuses more on the data side, while Galaxy and AnVIL focus more on a research project's workflow and analytics. Ideally, one can combine the two to receive the best from both worlds.

With Sherlock, if more advanced analytics is needed, one can easily generate custom data sets in standard formats, consumable by Galaxy or AnVIL tools (e.g. JSON, CSV or ORC). The results of the analytical workflows can then be imported to Sherlock again, enabling users to easily access this data and run ad hoc queries on them. In summary, when someone needs to run the same workflow/pipeline many times, then Galaxy and AnVIL are the better options, because they provide workspaces where workflows can be executed. On the other hand, when someone needs to work with more extensive databases with the same data format and wants to run multiple analytical queries to answer biological questions, then Sherlock would be the better option because this platform focuses on storing, manipulating, building and extending upon existing datasets.

We envision a more general data access mechanism with Sherlock as it uses industry-standard formats (such as JSON and ORC). It is important to note that with Sherlock, one can work with more project-specific and more customizable data. In the Sherlock platform, users can process and integrate data with their own needs. Sherlock is not database specific and it is flexible to research group-specific needs. For example, Sherlock is perfect when a group works with only a certain number of distinct or bespoke data types and data formats.

Since we made Sherlock, plenty of similar platforms have appeared worldwide, but none of these solutions is explicitly designed for computational biologists. One of them, for example, is the Qubole platform ( https://www.qubole.com). The Qubole platform also offers data storage and analytical query solutions, but operating the Sherlock platform is cheaper. Usually, research groups have their own servers or at least have access to one or more, and they have the opportunity to have data storage solutions. Having a server enables one to set up and use the platform itself because all the source code is freely available on GitHub and can be customized to the user's requirements. In this case, one has to pay only for the data storage space. Although Sherlock is compatible with any cloud provider, like Amazon AWS or Google Cloud, it was mainly developed and configured to the storage solutions of Digital Ocean.

To specifically support computational biologists, Sherlock contains specific database loader scripts that the users can use to create and upload the specific file formats to the Data Lake. We are constantly upgrading the already included datasets in our Data Lake. We also provide new database loader scripts in the Github repository (to extend interaction and expression data) and different mapping scripts. We will also develop different converting scripts to easily handle Sherlock compatibility with other file formats, such as TSV (tab-separated value) or CSV (comma-separated value).

Sherlock has a lot of valuable features, which are the following:

- Store all datasets in redundant and organized cloud storage
- Convert all datasets to standard, optimized file formats
- Execute analytical queries on top of data files
- Share datasets among different teams/projects
- Generate operational datasets for particular services or collaborators
- Provides a storage solution for big data computational biology projects

We intend to regularly update Sherlock's loader scripts to ensure compatibility with commonly used biological databases in the future and cover as many interactions and expression databases as possible. Furthermore, we plan to improve our source code to make more detailed documentation. Right now, updating the included databases in the Data Lake is a manual process, but we also would like to automate this with a script which can handle all of the updates at once. We would also like to include more common and general computational biology examples in the repository. To aid this, we are developing tutorials and extending the use cases of how Sherlock can be deployed and utilized for research projects. Our main goal is to disseminate the Sherlock platform widely, and we aim to enable computational and systems biologists to manage their large-scale datasets more effectively.

## Conclusion

Sherlock provides an open-source platform empowering data management, analytics, and collaboration through modern big data technologies. Utilizing the dockerization of Presto and the Hive Metastore, Sherlock is not only powerful but also a flexible and fast solution to store and analyze large biological datasets effectively and efficiently. Sherlock can be used to execute queries on top of a Data Lake, where all the data is stored in a ‘folder-like’ structure, providing the added benefit of well-defined folder structures, which helps and encourages correct data management of biological data. With a scalable query engine and ORC format, Sherlock can run SQL queries faster than other solutions, which can significantly reduce the lead time of the research project. In conclusion, Sherlock provides a ‘plug and play’ state-of-the-art data storage platform for large biological datasets via repurposing concepts and open source tools created by large software companies.

## Data availability

All data underlying the results are available as part of the article and no additional source data are required.

## Software availability

Software available from: https://earlham-sherlock.github.io/

Source code available from: https://github.com/earlham-sherlock/earlham-sherlock.github.io

Archived source code available from: http://doi.org/10.5281/zenodo.4738516 ( Bohar *et al.*, 2021)

License: MIT
