# Supplementary material for: Sherlock: an open-source data platform to store, analyze and integrate Big Data for computational biologists
Source: F1000Res. 2023 Jan 12;10:409. Originally published 2021 May 21. [Version 3] doi: 10.12688/f1000research.52791.3 (PMC9731172; doi:10.12688/f1000research.52791.3)
Supplement: Peer review history

# Peer review: Reviewer response for version 2

**DOI:** 10.5256/f1000research.136593.r147162

**Published:** 2022-12-06

**Authors:** Nadezhda T. Doncheva

**Affiliations:** 1 Novo Nordisk Foundation Center for Protein Research, University of Copenhagen, Copenhagen, Denmark

In the revised version of the article, the authors have very thoroughly addressed the comments of both reviewers. The flow of the article has benefited from restructuring the Implementation section, the added instructions related to setting up and using the Sherlock platform will be very helpful for its future users, and the Discussion section nicely puts Sherlock in the context of other tools used in the field. With this, the Sherlock platform can become an important addition to the set of tools used by computational biologists, especially those who work a lot with public datasets and datasets from collaborators.

 I have a minor remark related to use case 1, which has been changed in the revised version of the article. In the current version, Figure 8 only contains two tables and the last paragraph still refers to the protein identifiers expressed in the brain although this part is not covered by the example and query anymore. I personally found the previous version of use case 1 and the corresponding Figure 5 more interesting and relevant. Thus, I would recommend using the example with the three tables (the last one with the tissue information) if possible and, if that is considered too complex, then it would make sense to fix the last paragraph in this section to reflect the new query and figure.  

 There are also a few “typos” that could be easily fixed:

- Page 11, first column, third paragraph: “ *which version 2021_03*” should probably be “with”, but also can just be “version 2021_03”.
- Page 12, first column, second paragraph: the sentence that starts with “ *With Sherlock, the user can quickly*…” has "quickly" repeated twice.
- Page 12, second column, second paragraph: there is a “ *ref3*” in the middle of the white space that probably does not belong there.
- Page 13, second column, first paragraph: the sentence “ *which enables to answer simple*…” seems to be missing a word in the middle, I assume it should be “which enables users/computational biologists/…”.

Are the conclusions about the tool and its performance adequately supported by the findings presented in the article?

Yes

Is the rationale for developing the new software tool clearly explained?

Partly

Is the description of the software tool technically sound?

Yes

Are sufficient details of the code, methods and analysis (if applicable) provided to allow replication of the software development and its use by others?

Partly

Is sufficient information provided to allow interpretation of the expected output datasets and any results generated using the tool?

Yes

Reviewer Expertise:

Computational and network biology, development of software tools, analysis and integration of large datasets

I confirm that I have read this submission and believe that I have an appropriate level of expertise to confirm that it is of an acceptable scientific standard.

# Response

**Published:** 2022-12-21

**Authors:** Tamás Korcsmáros

**Affiliations:** Earlham Institute, UK

We thank the reviewers for their feedback on our manuscript and the constructive comments. The comments and questions led to an appropriate revision of the manuscript.

 We thank the Reviewer for the general comments and the approval of the second version of the manuscript. We also really appreciate the suggestions about Use Case 1 and minor typos. We changed the Use Case 1 accordingly and also fixed the typos in the manuscript.

# Peer review: Reviewer response for version 2

**DOI:** 10.5256/f1000research.136593.r147161

**Published:** 2022-09-06

**Authors:** John H. Morris

**Affiliations:** 1 Resource for Biocomputing, Visualization and Informatics, Department of Pharmaceutical Chemistry, University of California, San Francisco, San Francisco, CA, USA

I appreciate the authors' modifications in response to the reviewers' input and believe the resulting article to now be appropriately targeted to computational biologists with knowledge of SQL and basic database theory. I believe the tool will be useful as an engine for integrating local data sets into public data for further computational analysis.

Are the conclusions about the tool and its performance adequately supported by the findings presented in the article?

Partly

Is the rationale for developing the new software tool clearly explained?

Partly

Is the description of the software tool technically sound?

Yes

Are sufficient details of the code, methods and analysis (if applicable) provided to allow replication of the software development and its use by others?

Yes

Is sufficient information provided to allow interpretation of the expected output datasets and any results generated using the tool?

Yes

Reviewer Expertise:

computational biology

I confirm that I have read this submission and believe that I have an appropriate level of expertise to confirm that it is of an acceptable scientific standard.

# Response

**Published:** 2022-12-21

**Authors:** Tamás Korcsmáros

**Affiliations:** Earlham Institute, UK

We thank the reviewers for their feedback on our manuscript and the constructive comments. The comments and questions led to an appropriate revision of the manuscript.

 We thank the Reviewer for approving the second version of the paper. We also thank you very much for all of the constructive comments that helped us to improve the manuscript.

# Peer review: Reviewer response for version 1

**DOI:** 10.5256/f1000research.56112.r85970

**Published:** 2021-06-29

**Authors:** Nadezhda T. Doncheva

**Affiliations:** 1 Novo Nordisk Foundation Center for Protein Research, University of Copenhagen, Copenhagen, Denmark

In this article, the authors present an open-source platform called Sherlock, which can be very useful for computational biologists working with a lot of different datasets within one or several projects. The platform is designed to make use of well-known resources and concepts such as Docker, and PrestoDB. Sherlock is easily extendable and can be used both on a local computer or in connection with a cloud storage solution. Once Sherlock is properly set up and the needed data sets imported and formatted, the data can be integrated and analyzed using SQL queries as shown in the three simple use cases.  

 Sherlock can become a great addition to the suite of tools used by computational biologists, especially when it comes to bigger projects involving a lot of public datasets as well as own data generated by collaborators. The article is nicely written and gives a good overview of the Sherlock platform as well as possible use cases. However, it can be further improved in three main aspects as detailed below: making clear who is the target audience, improving the documentation about setting up Sherlock, and improving the flow of the Implementation and Operation section.

 Both the title and abstract mention that Sherlock is designed for biologists and only on page 6, the text mentions computational biologists specifically. However, both from the description of the setup and the specific use cases, it seems to me that the actual target audience should be clearly stated as “computational biologists”. It is worth considering changing the “for biology” part in the title since it is not very clear or specific as it is now.  

 One major concern is that neither the manuscript nor the online documentation seem to describe in enough detail how to set up the data needed for the three presented use cases. There is a nice deployment guide and the use case examples seem reasonable, but the step in-between is missing. The use cases assume that the user has an already configured Sherlock system with the necessary files stored, properly formatted and the right tables created, etc. I specifically checked the GitHub documentation, among others the “Loading … data to Sherlock” sections and I don’t think enough detail is given for a user like me to set it up (and I consider myself a computational biologist). I would need to know which raw data files from STRING were retrieved and stored in the raw zone, how were the JSON files generated that need to be copied to the landing zone, etc. I was able to find the scripts, which should help for this step ( https://github.com/earlham-sherlock/earlham-sherlock.github.io/tree/master/loaders) but they are also not described in enough detail, especially in connection with the use cases. So, what I think is needed is the following:

1. add a short description to each use case in the manuscript about which databases are needed to execute the example queries;
2. on GitHub, add a step-by-step tutorial for how to set up the specific databases/tables needed for the use cases. For use case 1, the tutorial should include information about how and from what data to create the tables “master.mapping”, ”master.tissues”, ”master.string_proteins”, while for use case 2 and 3, the needed tables are “master.bgee_2020_11_16” and “master.omnipath_2020_10_04”.

Another major comment is that the structure of the whole section “Implementation and Operation” can benefit from a bit of reorganization and section renaming. In the following, I will give some specific details and examples of how this can be done.

- The Overview section begins with a lengthy description of Docker and Docker Swarm and only gives an overview of the different components of Sherlock at the end of the section. The section will read much better if it begins with a high-level overview of the different components as it is partly done in the third paragraph (last one on page 3). This part can also refer to Figure 1. Then, each of the components can be explained in more or less detail (depending on the order and what follows in the next sections). For example, the end of paragraph one, which describes the scalability of Docker Swarm, also fits well with the last paragraph of the Overview section. This section could also be renamed to “Overview of the Sherlock platform”.
- In the Overview section, there seems to be a bit of confusion about the architecture & setup versus the usage workflow. The second paragraph starts with a sentence stating that “the first step of using Sherlock is to start a Docker Swarm”, while the third paragraph states that “the starting step for using Sherlock is to submit an SQL query to the Presto query engine”. I can see how both of these statements are true, but it will be easier to read if it is explained that one thing is part of the general, one-time setup of the system (which also fits more with the whole architecture), while the other one is the actual user workflow in a day-to-day usage (more relevant to the “Functionality of Sherlock” section).
- As a follow-up on that, it would be possible to move the whole description of Docker together with the “Query engine” and “Hive metastore” subsections into a section entitled “Architecture” or optionally “Architecture and setup” (instead of “Sherlock as a platform”).
- The last section about the functionality of Sherlock is currently very short and actually describes this day-to-day workflow. It could easily become part of the first overview section or else be extended by a few more details that do not fit so well into the Overview/Architecture sections.
- So, for the whole big section, the order would be “Overview of the Sherlock platform”, “Architecture (and setup)”, “Deployment”, “The Data lake – data storage” and optionally  “Functionality of Sherlock”.

In the following list, I have pointed out several minor things about the text or figures that can also be improved.

- The abstract says that “Sherlock is designed to analyse, process, query and extract the information from extremely complex and large datasets.” I think to be precise, it should say that it is designed “to facilitate/enable/allow users in analysing, processing, … “
- The description of Docker in the first paragraph of the Overview section could be shortened a bit (or moved to a more fitting place a bit later in the description).
- The legend of Figure 1 can be improved by describing the elements form left to right instead of right to left. It is not clear what a “Minio S3 server” is since it is not mentioned anywhere else in the manuscript. The first column of boxes in the Figure could also be surrounded by a bigger box and referred to as the user input (configuration) or something similar. Then it would be easier to refer to it.
- Page 4, second paragraph: “A Query Engines… are distributed” should be either singular or plural but not both.
- Page 4, third paragraph: the phrase “designed for working data” should probably be “designed for working with data” and “which held in” should be “which is held in”.
- Page 4, third paragraph: the sentence “Moreover it is particularly really useful to handle with structured data” can be rephrased for more clarity.
- Page 4, last paragraph: the sentence “With regards to Sherlock, Presto stores the metadata about the folders in the Data Lake and Hive metastore, which contains only the meta information about the different folders, which is in the Data Lake” also needs to be rephrased. There are too many “which” and it is not clear what refers to what and what information is actually contained/known to Presto and Hive metastore.
- Figure 2 does not look very nice in the PDF as opposed to the online version on GitHub, which has nicer formatting and quality. I would recommend replacing the figure in the PDF with the online version and making sure it still looks fine.
- Page 5, last paragraph: “on a local machine, on the cloud and the data…” should probably be “on a local machine and on the cloud and that the data…”.
- Page 5, last paragraph: the semicolon in the sentence “the most common Data Lake solutions;” could be replaced by a colon and “and with the Simple…” by “and Simple …”.
- Page 6, first paragraph of “Functionality of Sherlock”: how should the last sentence be understood? Does the user execute a “in-memory distributed query” or is this done also by the Presto engine?
- Page 6, last paragraph: the comma in “contain specific, interaction, …” is unnecessary.
- Page 7, there is an issue with the Omnipath reference in the table, it looks different and leads to a log-in page in the online version of the article.
- Page 7, first sentence “…we will outline three different use cases on how Sherlock can be used and what we can use it for” could be rephrased to sound better. Maybe just “… we will outline three different use cases of Sherlock”.
- Page 9, second paragraph: should this sentence “to enrich a network with interaction data” rather say “to retrieve interaction data for a gene list”?
- Figure 6: The two actions “Enrich with interconnections” and “Enrich with first neighbors” are repeated. I think it is enough to state this once below the arrow since it nicely describes the actual process. The picture before and after the arrow could have some other labels, but it is also fine if they do not have their own label.
- Page 10, second paragraph: the sentence “it contains specific database loader scripts which they are able to create and upload specific file formats to the Data Lake” needs some revising to make clear who is “they”. Maybe it should say “which the users are able to use to upload or can create themselves…”.
- Page 10, third paragraph: “followings” should be “following”.
- Page 10, last paragraph: there is probably a stop (sentence end) missing between “structure” and “providing” in “a ‘folder-like’ structure providing the added…”.
- I am not able to see the images in the html version of the article in Firefox on Mac, I can only see a name (f1db3fb0-55ef-43b8-9ea8-7f6486a79c9a_figure1.gif). If I right-click and try to see it in another tab, I see some XML code that says Access denied. I checked and I don’t have the same issue with other articles.

Are the conclusions about the tool and its performance adequately supported by the findings presented in the article?

Yes

Is the rationale for developing the new software tool clearly explained?

Partly

Is the description of the software tool technically sound?

Yes

Are sufficient details of the code, methods and analysis (if applicable) provided to allow replication of the software development and its use by others?

Partly

Is sufficient information provided to allow interpretation of the expected output datasets and any results generated using the tool?

Yes

Reviewer Expertise:

Computational and network biology, development of software tools, analysis and integration of large datasets

I confirm that I have read this submission and believe that I have an appropriate level of expertise to confirm that it is of an acceptable scientific standard, however I have significant reservations, as outlined above.

# Response

**Published:** 2022-07-21

**Authors:** Tamás Korcsmáros

**Affiliations:** Earlham Institute, UK

**Response to Reviewers for Manuscript ID: f1000research.52791.1**

 We thank the reviewers for their feedback on our manuscript and the constructive comments. The comments and questions led to a significant and appropriate revision of the manuscript.

***Answers for reviewer Nadezhda T. Doncheva***

*In this article, the authors present an open-source platform called Sherlock, which can be very useful for computational biologists working with a lot of different datasets within one or several projects. The platform is designed to make use of well-known resources and concepts such as Docker, and PrestoDB. Sherlock is easily extendable and can be used both on a local computer or in connection with a cloud storage solution. Once Sherlock is properly set up and the needed data sets imported and formatted, the data can be integrated and analyzed using SQL queries as shown in the three simple use cases.*

*Sherlock can become a great addition to the suite of tools used by computational biologists, especially when it comes to bigger projects involving a lot of public datasets as well as own data generated by collaborators. The article is nicely written and gives a good overview of the Sherlock platform as well as possible use cases. However, it can be further improved in three main aspects as detailed below: making clear who is the target audience, improving the documentation about setting up Sherlock, and improving the flow of the Implementation and Operation section.*

**We thank the Reviewer for these general comments and the summary about the manuscript. We also really appreciate the suggestions about the three main aspects.**

*Both the title and abstract mention that Sherlock is designed for biologists and only on page 6, the text mentions computational biologists specifically. However, both from the description of the setup and the specific use cases, it seems to me that the actual target audience should be clearly stated as “computational biologists”. It is worth considering changing the “for biology” part in the title since it is not very clear or specific as it is now.*

**Thank you very much for raising it, it is a good point. We agree with the Reviewer's view, and modified the manuscript throughout to reflect that Sherlock is primarily for computational biologists.**

*One major concern is that neither the manuscript nor the online documentation seem to describe in enough detail how to set up the data needed for the three presented use cases. There is a nice deployment guide and the use case examples seem reasonable, but the step in-between is missing. The use cases assume that the user has an already configured Sherlock system with the necessary files stored, properly formatted and the right tables created, etc. I specifically checked the GitHub documentation, among others the “Loading … data to Sherlock” sections and I don’t think enough detail is given for a user like me to set it up (and I consider myself a computational biologist). I would need to know which raw data files from STRING were retrieved and stored in the raw zone, how were the JSON files generated that need to be copied to the landing zone, etc. I was able to find the scripts, which should help for this step (https://github.com/earlham-sherlock/earlham-sherlock.github.io/tree/master/loaders) but they are also not described in enough detail, especially in connection with the use cases. So, what I think is needed is the following:*

*add a short description to each use case in the manuscript about which databases are needed to execute the example queries.*

*On GitHub, add a step-by-step tutorial for how to set up the specific databases/tables needed for the use cases. For use case 1, the tutorial should include information about how and from what data to create the tables “master.mapping”, ”master.tissues”, ”master.string_proteins”, while for use case 2 and 3, the needed tables are “master.bgee_2020_11_16” and “master.omnipath_2020_10_04”.*

**Thank you very much for your advice, these are really good and useful points. We have added the suggested clarifications for the github repository and for the Use Cases section in the revised manuscript as well.**

*Another major comment is that the structure of the whole section “Implementation and Operation” can benefit from a bit of reorganization and section renaming. In the following, I will give some specific details and examples of how this can be done.*

*The Overview section begins with a lengthy description of Docker and Docker Swarm and only gives an overview of the different components of Sherlock at the end of the section. The section will read much better if it begins with a high-level overview of the different components as it is partly done in the third paragraph (last one on page 3). This part can also refer to Figure 1. Then, each of the components can be explained in more or less detail (depending on the order and what follows in the next sections). For example, the end of paragraph one, which describes the scalability of Docker Swarm, also fits well with the last paragraph of the Overview section. This section could also be renamed to “Overview of the Sherlock platform”.*

**Thank you for bringing this to our attention, we have now improved this section and made the suggested restructuring changes.**

*In the Overview section, there seems to be a bit of confusion about the architecture & setup versus the usage workflow. The second paragraph starts with a sentence stating that “the first step of using Sherlock is to start a Docker Swarm”, while the third paragraph states that “the starting step for using Sherlock is to submit an SQL query to the Presto query engine”. I can see how both of these statements are true, but it will be easier to read if it is explained that one thing is part of the general, one-time setup of the system (which also fits more with the whole architecture), while the other one is the actual user workflow in a day-to-day usage (more relevant to the “Functionality of Sherlock” section).*

**Thank you for raising it as well, we have fixed these inaccuracies. In the Use Cases section, we added a general set up guide of the platform and the Data Lake. After that, we introduce how to run basic analytical SQL queries in different use cases.**

*As a follow-up on that, it would be possible to move the whole description of Docker together with the “Query engine” and “Hive metastore” subsections into a section entitled “Architecture” or optionally “Architecture and setup” (instead of “Sherlock as a platform”).*

**Thank you for your suggestion. We changed the section names and structure accordingly.**

*The last section about the functionality of Sherlock is currently very short and actually describes this day-to-day workflow. It could easily become part of the first overview section or else be extended by a few more details that do not fit so well into the Overview/Architecture sections.*

**Thank you, this is a good idea. We created a new subsection for the whole docker part and also extended it with more details.**

*So, for the whole big section, the order would be “Overview of the Sherlock platform”, “Architecture (and setup)”, “Deployment”, “The Data lake – data storage” and optionally  “Functionality of Sherlock”.*

**Thank you for your advice. We have restructured the whole Implementation and Operation section based on your suggestions, and also we fixed the minor points that you raised.**

 Thank you very much for all of the constructive comments that helped us to improve the manuscript.

# Peer review: Reviewer response for version 1

**DOI:** 10.5256/f1000research.56112.r85971

**Published:** 2021-06-17

**Authors:** John H. Morris

**Affiliations:** 1 Resource for Biocomputing, Visualization and Informatics, Department of Pharmaceutical Chemistry, University of California, San Francisco, San Francisco, CA, USA

In this article, the authors describe a system, Sherlock, for storing and accessing data using SQL. The system described is flexible, and uses modern approaches to the standard ETL (extract, transform, load) processes common in a data warehouse solution. The system seems like it would be extremely useful for a narrow set of use cases, somewhat useful for a larger set of use cases, and of questionable utility for most computational biology use cases. 

 Sherlock's main utility, it seems to me, is for facilities that have large repositories of self-generated data. In that circumstance, the overhead of learning the column names, appropriate foreign keys, and the appropriate SQL syntax to manipulate the data would be well-warranted, and I would imagine in those circumstances, the data could be stored locally on some fast media that would provide significant performance advantages, particularly given the distributed nature of presto. 

 I can also see Sherlock being useful in circumstances where there is a relatively static pipeline that operates on data sources that are more commonly updated. In this circumstance, the ability to have scripts to stage the data and use a common SQL syntax that doesn't change would be useful.

 In other circumstances, I'm less convinced. First, while I appreciate the value of the multiple-level folder structure (Raw Zone --> Landing Zone --> Master Zone --> Project Zone) that results in a number of copies of databases that could be extremely large. The examples given are relatively small, but consider downloading TrEMBL, which is 132 Gb compressed, and reprocessing that to get at least 3 copies (assuming one would subset it for the Project Zone). The scary part is that TrEMBL is small by modern standards -- image datasets are significantly larger. Second, while I agree that for software developers, SQL is a well-known and convenient query language, it is certainly non-trivial to construct efficient queries and not the best interactive tool for exploring data sets. Further, there is no attempt to fit this tool into the more broad set of tools commonly used by computational biologists. For example, why would I use Sherlock for scRNA-seq analysis if I have access to Galaxy or AnVIL?

 So, not to be totally negative, I think Sherlock has a place in the tool suite for computational biology, but I don't think that place is well articulated in the article. My recommendations for improving the article are:

1. The second sentence in the introduction talks about working on bespoke datasets, which I suspect is the crux of Sherlock. The rest of the article seems to ignore that, and even the use cases focus purely on public data. This weakens the case for Sherlock. The use cases are trivially (OK, so relatively) easy using a combination of python, perhaps with pandas, data files and web services. The power of Sherlock is the ability to *integrate* public databases with bespoke databases, but that really isn't highlighted sufficiently. That needs to be the focus on the user cases, I think.
2. Discuss Sherlock in the context of other tools (not technologies) that support computational biology, such as Galaxy and AnVIL as two examples. When would I use those tools? When would I use Sherlock? Why is Sherlock better in that case?
3. Be clearer as to the target user. In several cases, the article states that this is "designed specifically for biologists". I actually don't think that's correct. It's designed for computational biologists or bioinformaticians with a very strong computational background. I don't know many bench biologists who would be able to formulate some of the SQL queries, even if it was easy to figure out all of the column names. I don't *think* the authors believe that SQL is the user interface for the system as much as an API for accessing the data, but that also wasn't clear in the text.
4. The role of Hive is somewhat obscure to me. I got that it stores the metadata, but *what* metadata? Is that where I can look up the schema to get the column names for joins and projections? In the use cases, it seems like there were several jumps in logic. Where does the user figure out that the ensemble ID in the STRING database is stored as "ens_id"? How well exposed is the schema for each of the tables in the data lake and how does a user (who didn't happen to be the one who imported the data) figure that out?
5. One of the main points in the article is the potential for high performance due to the parallelism of presto, but there were no performance numbers offered, either for the ETL phase or for the queries themselves.
6. Finally, as a more minor point, there are several places where the manuscript could use a bit of an edit pass. For example, on page 2 it states "A Query Engines, for example " shouldn't be plural.

Sherlock clearly represents a *lot* of work, and I do believe that it could be valuable to the appropriate audience and that it should be published.  My take on the manuscript is that it's not clear who it is written for. If it's written for a computer scientist, then information about performance metrics and local vs. cloud storage trade-offs and scalability, etc., are missing. If it's written for the computational biologist, then it's not really necessary to address the trade-offs between docker swarm and kubernetes, or why SQL can be considered composed of three main sub-languages, but it is important to explain why Sherlock is better than Galaxy or AnVIL and a little more detail on how the user figures out things like column names, etc.

 Sorry if this comes across as overly negative. I honestly do see how much work is involved and I can imagine where it might be extremely useful in some environments, but that just doesn't come across clearly in this submission.

Are the conclusions about the tool and its performance adequately supported by the findings presented in the article?

Partly

Is the rationale for developing the new software tool clearly explained?

Partly

Is the description of the software tool technically sound?

Yes

Are sufficient details of the code, methods and analysis (if applicable) provided to allow replication of the software development and its use by others?

Yes

Is sufficient information provided to allow interpretation of the expected output datasets and any results generated using the tool?

Yes

Reviewer Expertise:

computational biology

I confirm that I have read this submission and believe that I have an appropriate level of expertise to confirm that it is of an acceptable scientific standard, however I have significant reservations, as outlined above.

# Response

**Published:** 2022-07-21

**Authors:** Tamás Korcsmáros

**Affiliations:** Earlham Institute, UK

**Response to Reviewers for Manuscript ID: f1000research.52791.1**

 We thank the reviewers for their feedback on our manuscript and the constructive comments. The comments and questions led to a significant and appropriate revision of the manuscript.

***Answers for reviewer John H. Morris***

*In this article, the authors describe a system, Sherlock, for storing and accessing data using SQL. The system described is flexible, and uses modern approaches to the standard ETL (extract, transform, load) processes common in a data warehouse solution. The system seems like it would be extremely useful for a narrow set of use cases, somewhat useful for a larger set of use cases, and of questionable utility for most computational biology use cases.*

*Sherlock's main utility, it seems to me, is for facilities that have large repositories of self-generated data. In that circumstance, the overhead of learning the column names, appropriate foreign keys, and the appropriate SQL syntax to manipulate the data would be well-warranted, and I would imagine in those circumstances, the data could be stored locally on some fast media that would provide significant performance advantages, particularly given the distributed nature of presto. I can also see Sherlock being useful in circumstances where there is a relatively static pipeline that operates on data sources that are more commonly updated. In this circumstance, the ability to have scripts to stage the data and use a common SQL syntax that doesn't change would be useful.*

**We thank the Reviewer for these general comments and we agree with the specific use cases described by the Reviewer on where Sherlock could be useful.**

*In other circumstances, I'm less convinced. First, while I appreciate the value of the multiple-level folder structure (Raw Zone --> Landing Zone --> Master Zone --> Project Zone) that results in a number of copies of databases that could be extremely large. The examples given are relatively small, but consider downloading TrEMBL, which is 132 Gb compressed, and reprocessing that to get at least 3 copies (assuming one would subset it for the Project Zone). The scary part is that TrEMBL is small by modern standards -- image datasets are significantly larger. Second, while I agree that for software developers, SQL is a well-known and convenient query language, it is certainly non-trivial to construct efficient queries and not the best interactive tool for exploring data sets. Further, there is no attempt to fit this tool into the more broad set of tools commonly used by computational biologists. For example, why would I use Sherlock for scRNA-seq analysis if I have access to Galaxy or AnVIL?*

**We thank the Reviewer for this comment, which is really helpful as these were not properly covered in the original submission. Regarding the multi-level folder structure, we added a paragraph into the end of “The Data Lake - data storage” section to explain its usefulness further. Regarding the last part of this comment, we added paragraphs to the “Discussion” section of the revised manuscript.**

*So, not to be totally negative, I think Sherlock has a place in the tool suite for computational biology, but I don't think that place is well articulated in the article. My recommendations for improving the article are:*

*The second sentence in the introduction talks about working on bespoke datasets, which I suspect is the crux of Sherlock. The rest of the article seems to ignore that, and even the use cases focus purely on public data. This weakens the case for Sherlock. The use cases are trivially (OK, so relatively) easy using a combination of python, perhaps with pandas, data files and web services. The power of Sherlock is the ability to integrate public databases with bespoke databases, but that really isn't highlighted sufficiently. That needs to be the focus on the user cases, I think.*

**We would like to thank you for this comment and also thanks for the suggestions, we modified the manuscript accordingly. We added that part into the “Overview of the platform” section.**

*Discuss Sherlock in the context of other tools (not technologies) that support computational biology, such as Galaxy and AnVIL as two examples. When would I use those tools? When would I use Sherlock? Why is Sherlock better in that case?*

**Thank you for these important questions not yet addressed in the manuscript. We added new paragraphs about AnViL and Galaxy into the “Discussion” section to address these.**

Be clearer as to the target user. In several cases, the article states that this is "designed specifically for biologists". I actually don't think that's correct. It's designed for computational biologists or bioinformaticians with a very strong computational background. I don't know many bench biologists who would be able to formulate some of the SQL queries, even if it was easy to figure out all of the column names. I don't *think* the authors believe that SQL is the user interface for the system as much as an API for accessing the data, but that also wasn't clear in the text.

**Thank you for this comment as well. This is a valid point as indeed Sherlock was designed for computational biologists or data scientists. Therefore, we clarified it in the revised manuscript and updated the title as well.**

The role of Hive is somewhat obscure to me. I got that it stores the metadata, but *what* metadata? Is that where I can look up the schema to get the column names for joins and projections? In the use cases, it seems like there were several jumps in logic. Where does the user figure out that the ensemble ID in the STRING database is stored as "ens_id"? How well exposed is the schema for each of the tables in the data lake and how does a user (who didn't happen to be the one who imported the data) figure that out?

**Thank you for raising this. Hive is actually more of a technical part, which is used by PrestoDB. Sherlock stores metadata (columns structure) about the tables for Presto to query the files. For best practice, each user/user group needs to add a schema to describe what is inside the tables (wiki page, loader scripts, SQL editors). We have extended the manuscript to clarify these points in the “Functionality of Sherlock” section.**

*One of the main points in the article is the potential for high performance due to the parallelism of presto, but there were no performance numbers offered, either for the ETL phase or for the queries themselves.*

**Thank you for pointing out that this comparison was missing.  We added test measurement to the manuscript into the “The Data Lake - data storage” section.**

*Finally, as a more minor point, there are several places where the manuscript could use a bit of an edit pass. For example, on page 2 it states "A Query Engines, for example " shouldn't be plural.*

**Thank you for raising this, we reviewed and corrected the text now.**

Sherlock clearly represents a *lot* of work, and I do believe that it could be valuable to the appropriate audience and that it should be published.  My take on the manuscript is that it's not clear who it is written for. If it's written for a computer scientist, then information about performance metrics and local vs. cloud storage trade-offs and scalability, etc., are missing. If it's written for the computational biologist, then it's not really necessary to address the trade-offs between docker swarm and kubernetes, or why SQL can be considered composed of three main sub-languages, but it is important to explain why Sherlock is better than Galaxy or AnVIL and a little more detail on how the user figures out things like column names, etc.

**Thank you very much for this summary and the suggestions. We agree with the Reviewer's view. Accordingly, we modified the manuscript throughout to meet with the interest and needs of a computational biologist. In some cases, we kept some background information (such as trade-offs between docker swarm and kubernetes) but rephrased them in a restructured arrangement.**

*Sorry if this comes across as overly negative. I honestly do see how much work is involved and I can imagine where it might be extremely useful in some environments, but that just doesn't come across clearly in this submission.*

**Thank you very much for all of the constructive criticism and helping us to improve the manuscript.**
